# Integrated Comparative Genomics and Population Genomics Reveal the Genomic and Epigenomic Basis of Barley (*Hordeum vulgare*) Evolution and Domestication

**DOI:** 10.3390/plants15152341

**Published:** 2026-07-29

**Authors:** Wenhao Yue, Kangfeng Cai, Lei Liu, Yong Li, Shengguan Cai, Junmei Wang

**Affiliations:** 1 National Barley Improvement Center, Zhejiang Academy of Agricultural Sciences, Hangzhou 310021, China; yuewh@zaas.ac.cn (W.Y.); caikf@zaas.ac.cn (K.C.); liulei453@126.com (L.L.); liyongcl@163.com (Y.L.); 2 College of Agriculture and Biotechnology, Zhejiang University, Hangzhou 310058, China

**Keywords:** transposable element, methylation, domestication, three-dimensional (3D) genome architecture

## Abstract

Barley (*Hordeum vulgare*) is one of the earliest domesticated cereal crops and remains important for global feed, food, and malting industries. Although high-quality genomic resources are now available, its large repeat-rich genome has complicated genome-wide characterization of genomic features associated with barley evolution and domestication. Here, we integrated comparative genomics, methylomics, population genomics, and Hi-C analyses to investigate genome evolution and domestication in barley. Comparative analyses across representative grass species revealed that barley exhibits lineage-specific genome expansion associated with the extensive proliferation of long terminal repeat retrotransposons. Genome-wide DNA methylation analyses revealed distinct cytosine methylation landscapes in barley. Population genomic analyses of 291 wild and domesticated barley accessions revealed relatively slow linkage disequilibrium decay and identified 243 candidate domestication-associated genes. These candidate genes were enriched in starch and sucrose metabolism pathways. Comparative Hi-C analyses of three barley accessions further uncovered differences in A/B chromatin compartments and topologically associating domain (TAD) organization despite their largely conserved gene content. In addition, the enrichment of DNA transposons and Copia retrotransposons at TAD boundaries suggests potential associations between specific TE classes and higher-order chromatin organization. Together, these complementary analyses connect genome expansion, epigenetic regulation, domestication-associated selection, and chromatin organization into a unified framework for understanding barley genome evolution and domestication.

## 1. Introduction

Most hereditary information in plants is encoded within their genomes. Comparative analyses of plant genomes provide fundamental insights into genomic evolution, speciation, and the emergence of species-specific genomic features that underpin environmental adaptability and shape plant diversity [1]. Comparative analyses among representative Poaceae species can provide an evolutionary framework for distinguishing lineage-specific genomic features of barley (*Hordeum vulgare*) from shared patterns across grass genomes. Transposable elements (TEs) are ubiquitous components of plant genomes and can relocate within genomes using a process known as transposition [2]. TEs are broadly classified into two major classes: DNA transposons, where DNA sequences mobilize using a cut-and-paste mechanism, and retrotransposons, which mobilize using a copy-and-paste mechanism requiring RNA intermediates and represent the predominant TE class in plant genomes [2,3]. The retrotransposons are further divided into long terminal repeat (LTR) retrotransposons, which largely contribute to plant genome size variations, and non-LTR retrotransposons [2,3]. Although TEs generate raw genetic variations and can facilitate phenotypic diversification during plant domestication and adaptation [4,5], they can also result in deleterious or even lethal mutations. To maintain genomic stability while preserving the capacity for rapid responses to environmental changes, plants employ reversible epigenetic mechanisms to silence TE activity [2]. DNA methylation, also known as 5-methylcytosine (5 mC), is an epigenetic modification that plays important roles in TEs, transcriptional regulation, and plant growth and development [6,7]. DNA methylation occurs in three sequence contexts: CG, CHG and CHH (H denotes A, T or C). DNA methylation influences the transcription of genes and TEs mainly in three patterns. Fully silenced genes and TEs are associated with high levels of methylated CG (mCG), mCHG, and mCHH. Gene body CG methylation alone is often linked to active transcription, whereas any other types of DNA methylation are mostly associated with transcription repression [2,6]. Widespread variations in DNA methylation exist among plant species, and different DNA methylation patterns reflect the evolution histories of plant species [8].

Plant genomes are spatially organized within the nucleus into complex three-dimensional (3D) chromatin architectures, which modulate gene transcription by allowing regulatory elements, far from target genes in the one-dimensional sequence, to approach target genes in spatial structures [9,10,11]. Plant chromatin architecture is generally organized into three hierarchical levels, ranging from megabase-scale compartments to kilobase-scale topologically associating domain (TAD) and chromatin loop [9,10,11]. A compartment regions are generally gene rich and transcriptionally active, whereas B compartment regions are enriched in TEs and repressed epigenetic modifications [9,10,11]. TADs are relatively local units, and the interactions within TADs are significantly stronger than those between different TADs [9,10,11]. Chromatin loops establish physical contacts between distant genomic regions, such as regulatory elements and promoters, to modulate gene expression [9,10,11]. Different chromatin structures contribute to cell type-specific gene expression patterns [11], and the chromatin landscape exhibits domestication-associated remodeling [12,13] and adaptation to environmental changes [14,15].

Domestication represents a major evolutionary process that has profoundly shaped plant genomes and agronomic traits. Through long-term artificial selection, crop domestication has resulted in characteristic phenotypic changes, collectively known as domestication syndrome [16,17], including reduced seed dispersal, altered plant architecture, and changes in reproductive traits. Barley (*Hordeum vulgare*) was one of the earliest domesticated cereal crops, having been domesticated approximately 10,000 years ago from its wild progenitor (*Hordeum spontaneum*) in the Fertile Crescent region [18]. During this process, strong selection pressures reduced genetic diversity in cultivated barley populations and generated detectable genomic signatures, including selective sweeps and changes in allele frequencies at domestication-associated loci [19]. Previous studies have identified several key genes underlying barley important domestication traits [20], such as spike row type [21] and grain dispersal [22]. The identification of positively selected genes has expanded our understanding of the genetic basis underlying barley domestication [23]. However, domestication is not solely driven by changes in protein-coding sequences. Transposable elements [4], epigenetic modifications [24], and higher-order chromatin organization [12] may also contribute to evolutionary diversification and phenotypic adaptation. Therefore, integrating information from genome sequence variation, repetitive elements, epigenetic landscapes, and chromatin organization provides an opportunity to gain a more comprehensive understanding of the molecular processes underlying barley genome evolution and domestication.

The self-pollinating nature, diploid genome, and rich germplasm resources of barley established it as a model species for basic research in the 20th century [25,26]. Nevertheless, the large genome of barley (approximately 4.5 Gb), characterized by more than 80% repeat elements and low recombination [27], posed significant challenges for whole-genome sequencing and assembly, resulting in the first report of chromosome-scale barley reference genome in 2017 [27]. This was considerably later than model species Arabidopsis (*Arabidopsis thaliana*) and rice (*Oryza sativa*). The rapid innovations of high-throughput sequencing technologies and decreasing costs advanced the qualities of barley reference genomes in recent years [28,29,30] and generated diverse omics resources, including whole-genome resequencing [28,30], methylation [31], and high-throughput chromosome conformation capture (Hi-C) datasets [28,32]. Together, these resources provide new opportunities to investigate different but complementary aspects of barley genome evolution and domestication from genomic, epigenomic, and chromatin perspectives. In this study, we integrated comparative genomics, methylomics, population genomics, and 3D chromatin analyses to characterize complementary aspects of barley genome evolution and domestication. Comparative genomic and methylome analyses were used to investigate lineage-specific genome expansion and the epigenetic landscapes associated with TE-rich genomes. Population genomic analyses were performed to identify candidate genomic regions potentially shaped by domestication selection, while comparative Hi-C analyses among three barley accessions were conducted to characterize variation in higher-order chromatin organization. Together, these analyses provide an integrated framework for characterizing genomic features associated with barley genome evolution and domestication.

## 2. Results

### 2.1. Comparative Genomic Analyses Reveal Lineage-Specific Features of the Barley Genome

**Figure 1:**
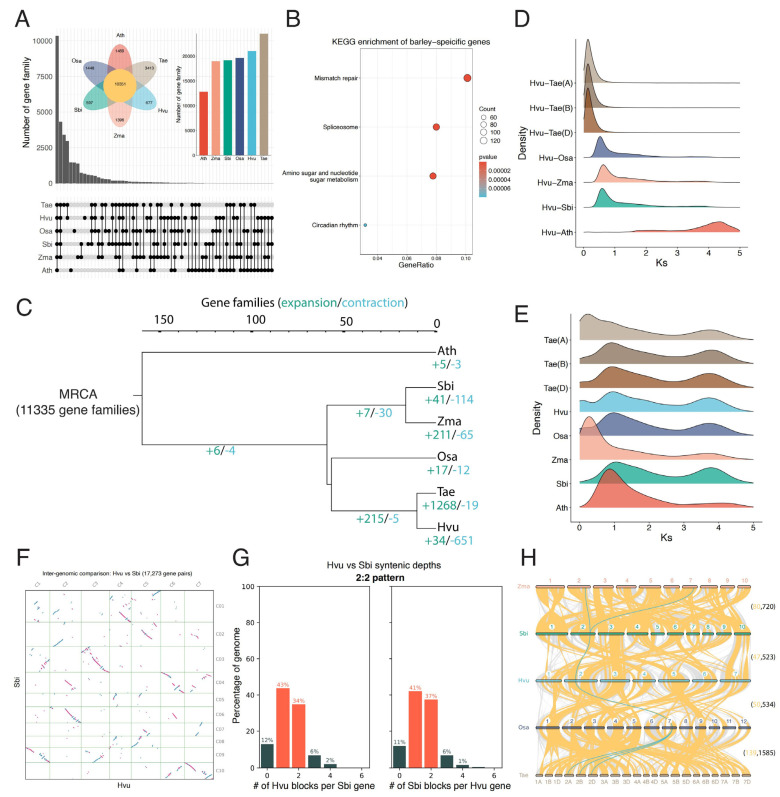
Comparative genomic analysis of barley, four representative grass species, and Arabidopsis. (**A**) Gene family clustering analysis. Inset images display the numbers of the common and species-specific gene families and the total numbers of gene families in each plant species. (**B**) KEGG analysis of barley-specific gene families. (**C**) Gene family expansion and contraction analysis. The phylogenetic tree of plant species was based on the concatenation of 2502 single copy genes. The green and blue colors mark the numbers of expanded and contracted gene families, respectively. MRCA, most recent common ancestor. (**D**,**E**) Estimations of species divergences between barley and other plant species (**D**) and whole-genome duplications within different plant species (**E**). (**F**,**G**) Dot plot analysis (**F**) and syntenic depth analysis (**G**) of genome-wide syntenic blocks between barley and sorghum. (**H**) The genome-wide syntenic analysis among different plant species. The numbers in the parentheses indicate large (>200 gene pairs, yellow) and small (5–200 gene pairs, black) syntenic blocks. Representative relationships among the plant species are denoted by blue lines. Abbreviation: Ath, *Arabidopsis thaliana*; Osa, *Oryza sativa*; Sbi, *Sorghum bicolor*; Zma, *Zea mays*; Hvu, *Hordeum vulgare*; Tae, *Triticum aestivum*.

To investigate the genomic evolution of barley, we obtained the genome assemblies (Table S1) of barley, Arabidopsis, and four representative grass species (sorghum, maize, rice, and wheat) and performed gene family clustering. A total of 32,207 gene families were identified among the six plant species (Table S2). Among them, 21,024 gene families were detected within barley, fewer than wheat (25,554 gene families) but more than Arabidopsis (12,803), sorghum (19,172), maize (18,936), and rice (19,618) (Figure 1A and Table S3). A total of 667 gene families comprising 10,447 genes were specific to barley. Kyoto Encyclopedia of Genes and Genomes (KEGG) enrichment results indicated that these genes were mainly involved in mismatch repair, spliceosome, amino sugar and nucleotide sugar metabolism, and circadian rhythm pathways (Figure 1B and Table S4). Consistently, the GO enrichment of the barley-specific genes was associated with primary cellular process such as RNA transcription, mismatch repair, and chromatin organization (Figure S1 and Table S5). Gene family expansion and contraction patterns were examined across the six plant species, and we identified 34 expanded gene families in barley, substantially fewer than the closely related species wheat (1268 gene family). In contrast, 651 gene family were contracted in barley, markedly more than wheat (19 gene family) (Figure 1C).

Species divergence was inferred based on the density distributions of synonymous nucleotide substitution (Ks) within syntenic blocks between barley and the five other plant species, with the wheat genome divided into A, B, and D subgenomes. These analyses indicated that barley diverged earliest from Arabidopsis, followed by divergence from the common ancestor of sorghum and maize, and subsequently from rice and wheat (Figure 1D and Figure S2A). Furthermore, polyploidization event analyses based on intra-genomic syntenic blocks revealed three peaks in barley and two peaks in other plant species (Figure 1E and Figure S2B; Table S6). The ancient Ks density peak (Ks ~ 4.0) among all species denoted the ancient whole-genome duplication among angiosperms [33,34] (Figure 1E), and the shared peak (Ks ~ 1.0) corresponded to the grass-specific rho whole-genome duplication [35] (Figure 1E). To determine whether the peak (Ks ~ 0.2) in barley represented a whole-genome duplication event, we carried out genomic comparisons between barley and sorghum species that have not experienced whole-genome duplication in recent 70 million years [36]. Genome-wide synteny (Figure 1F) and syntenic depth analysis (Figure 1G) indicated that barley did not undergo whole-genome duplication (WGD) or whole-genome triplication (WGT) after divergence from sorghum. Instead, the Ks peak (Ks ~ 0.2) represented the previously reported burst of recent gene duplications in Triticeae [37]. We then mapped the barley gene sequences onto the other plant species to infer their inter-genomic synteny and polyploidization events. More syntenic blocks were observed between barley and species that underwent recent WGD (maize, 866 syntenic blocks, 30 large blocks with gene pairs > 200) or hexaploidy species wheat (1436, 140) than other species such as rice (534, 50), sorghum (523, 47), and Arabidopsis (177, 0) (Figures S3–S5). Moreover, barley exhibited a 1:1 syntenic ratio with sorghum and rice, a 1:2 ratio with maize, and a 1:3 ratio with hexaploid wheat (Figure 1H and Figure S6–S8), consistent with known polyploidization histories [35]. Furthermore, barley exhibited good synteny with each subgenome of wheat in chromosome levels, except a large inversion in chromosome 4A (Figure S8). These results indicate that the barley genome did not undergo whole-genome polyploidization events after divergence from sorghum, but instead experienced recent segmental duplications. Given that genome size variation among plant species is frequently associated with lineage-specific accumulation of repetitive sequences, particularly transposable elements [3], we next investigated the contribution of TE dynamics to barley genome evolution.

### 2.2. LTR Retrotransposon Proliferation Contributes to Barley Genome Expansion

**Figure 2:**
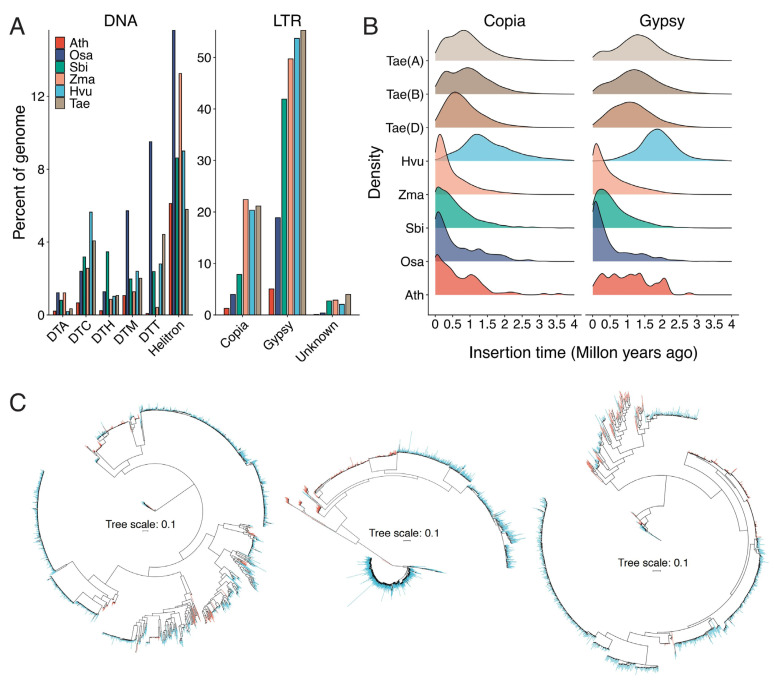
LTR retrotransposon proliferation is associated with barley genome expansion. (**A**) The contents of transposable elements. DNA transposons were classified into Helitron and five major superfamilies [40], including DTA (hAT), DTC (CATA), DTH (PIF/Harbinger), DTM (Mutator), and DTT (Tc1/Mariner). (**B**) Estimation of insertion times of Copia and Gypsy transposons. (**C**) Phylogenetic tree of intact Copia transposons (**left**), chromovirus type Gypsy transposons (**middle**), and non-chromovirus type Gypsy transposons (**right**) of barley and rice. The phylogenetic trees include 1646 Copia transposons of barley and 318 of rice (**left**), 947 chromovirus type Gypsy transposons of barley and 414 of rice (**middle**), and 2360 non-chromovirus type Gypsy transposons of barley and 500 of rice (**right**). Abbreviation: Ath, *Arabidopsis thaliana*; Osa, *Oryza sativa*; Sbi, *Sorghum bicolor*; Zma, *Zea mays*; Hvu, *Hordeum vulgare*; Tae, *Triticum aestivum*.

TEs constitute major components of plant genomes and play important roles in genomic evolution [38,39]. To characterize the potential contributions of TEs to genome evolution in barley, we annotated the repeat sequences in the genomes of barley and the five other plant species using a uniform annotation pipeline. Repeat sequences accounted for 88.34% of barley genome (genome size ~ 4.26 Gb), comparable to the proportion in maize (85.43%, 2.11 Gb) and wheat (88.37%, 14.23 Gb), but higher than sorghum (68.91%, 683.65 Mb), rice (51.12%, 373.25 Mb), and Arabidopsis (14.31%, 119.15 Mb) (Table S7). These results suggest that the repeat sequence content increased with genome size. We further focused on DNA transposons and long terminal repeat retrotransposons (LTR-RTs). LTR-RTs, particularly Gypsy elements (Figure 2A), constituted the major fraction of repeat sequences across all grass plant species, accounting for 80.83% proportion in barley, comparable to maize (80.21%) and wheat (84.12%), but higher than sorghum (75.81%) and rice (43.62%) (Figure 2A and Table S7).

To characterize transposable element amplification dynamics, we identified the full-length LTR-RTs in each genome and estimated their insertion times. In barley, LTR-RT insertions showed a continuous accumulation pattern, with distinct insertion peaks at approximately 1.0–1.5 and 1.8–2.2 million years ago (Mya) for Copia and Gypsy elements, respectively (Figure 2B). These peaks were older than those observed in the other four grass species (Figure 2B and Figure S9). The LTR-RT expansions in barley after divergence from rice were further supported by the phylogenetic tree of full-length LTR-RTs (Figure 2C). These results revealed that extensive LTR-RT proliferation contributed substantially to the large genome size of barley. Because uncontrolled TE activity can threaten genome stability, plants have evolved epigenetic mechanisms, particularly DNA methylation, to regulate TE activity [2]. We therefore compared DNA methylation landscapes among plant species to investigate the epigenetic regulation associated with TE-rich barley genomes.

### 2.3. Comparative DNA Methylation Analyses Reveal TE-Associated DNA Methylation Landscapes in Barley

**Figure 3:**
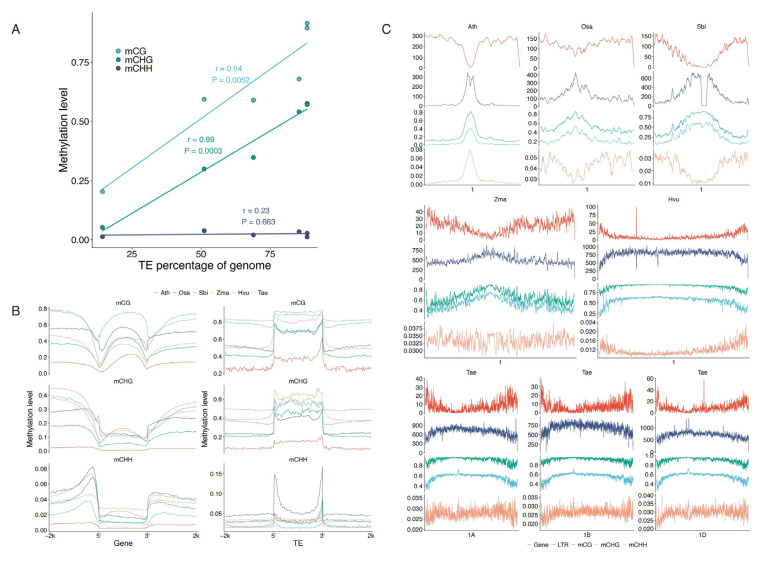
Distinct DNA methylation patterns among barley, four representative grass species, and Arabidopsis. (**A**) Correlation between DNA methylation levels and TE content. (**B**) Methylation patterns of mCG, mCHG, and mCHH across the genomic regions ranging from 2 kb upstream to 2 kb downstream of genes (**left**) and TEs (**right**). (**C**) Distribution of genes, LTRs, and DNA methylation levels of mCG, mCHG, and mCHH along chromosome 1 in different plant species, using 1 Mb sliding windows with 200 kb steps. Abbreviation: Ath, *Arabidopsis thaliana*; Osa, *Oryza sativa*; Sbi, *Sorghum bicolor*; Zma, *Zea mays*; Hvu, *Hordeum vulgare*; Tae, *Triticum aestivum*.

Plants repress TE activity to maintain genome stability using various chromatin modifications, such as cytosine DNA methylation [2]. Given the substantial variations of TE content among surveyed plant species, we expected pronounced differences in the DNA methylation patterns. We obtained publicly available leaf whole-genome bisulfite sequencing (WGBS) datasets from the comparative plant species (Table S1; >26-fold for all species), and quantified the DNA methylation levels at single-nucleotide resolution. It should be noted that the WGBS data of barley was obtained from a wild accession Rossa166 [31], because publicly available methylome data from domesticated barley were unavailable. Genome-wide DNA methylation levels showed a positive association with genomic TE proportions (Figure S10). Moreover, methylation levels of mCG and mCHG were positively correlated with genomic TE proportions, whereas mCHH exhibited no significant correlation (Figure 3A). We next examined the methylation levels of different contexts in the distinct genomic features. For genes, the methylation levels of different contexts decreased at the boundaries of genes across all species relative to flanking regions. The methylation of mCG within gene body returned to the flanking levels, whereas mCHG and mCHH remained at low levels throughout gene body regions (Figure 3B). Additionally, barley exhibited the highest methylation levels of mCG and lowest methylation levels of mCHH within gene regions among grass species examined (Figure 3B). For TEs, methylation levels of different contexts increased at the boundaries in all species relative to flanking regions and remained at high levels within the TE body regions (Figure 3B). Similar to gene regions, barley exhibited almost the highest methylation levels of mCG and lowest methylation levels of mCHH in the TE regions among grass species (Figure 3B). Furthermore, chromosome-scale analyses revealed that methylation levels of mCG and mCHG positively correlated with LTR-RT density and negatively with gene density across all species, whereas mCHH exhibited distinct correlation patterns among different plant species (Figure 3C, Figures S11 and S20). Notably, the methylation levels of mCHH in barley positively correlated with gene density (correlation coefficient r = 0.72; *p* < 2.2 × 10^−16^) and negatively correlated with LTR-RT density (r = −0.54; *p* < 2.2 × 10^−16^) (Figure S20), a pattern also observed in rice and sorghum. In contrast, no strong correlation of mCHH with the gene or LTR-RT density (r < 0.2; *p* < 0.001) were detected in maize and wheat (Figure S20). These results indicate that DNA methylation patterns are closely associated with TE content and distribution in the barley genome. Beyond lineage-specific genome evolution and epigenetic regulation, domestication represents another major evolutionary force that has shaped the genetic diversity of barley. We therefore next investigated genomic signatures potentially associated with domestication selection.

### 2.4. Population Genomic Analyses Identify Candidate Domestication-Associated Genes in Barley

**Figure 4:**
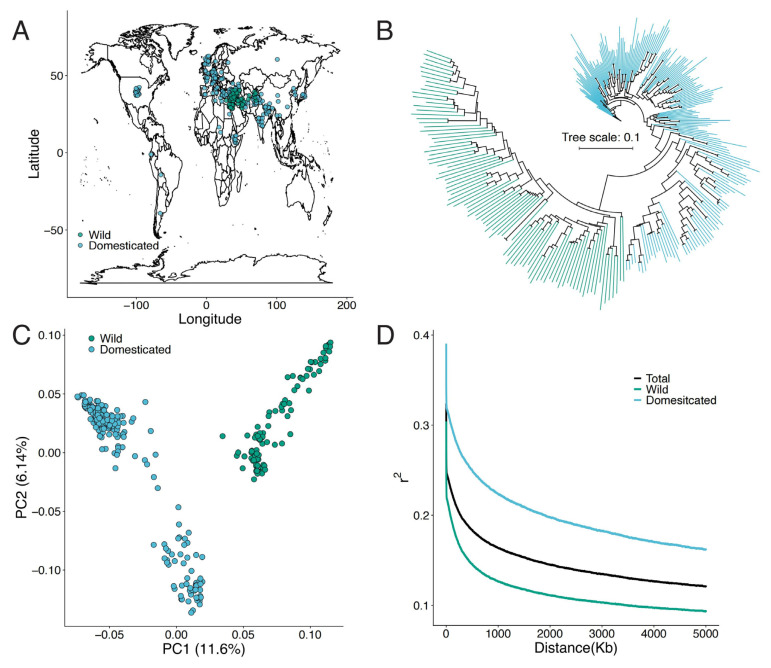
Geographic distribution, population structure, and decay of linkage disequilibrium (LD) of resequencing barley accessions. (**A**) The global geographic distribution of resequencing accessions. (**B**) The phylogenetic tree of resequencing accessions based on the whole-genome SNPs. (**C**) Principal component analysis of resequencing accessions in the first and second dimensions. (**D**) Decay of LD in whole, wild, and domesticated populations. The whole, wild, and domesticated populations are marked with black, green, and blue colors, respectively.

The selective sweeps or selected genes during barley domestication have been investigated using exome sequencing [19,41], which targeted approximately 60 Mb of coding sequences of barley genome [42]. To characterize domestication-associated genomic signatures at the genome-wide scale, we analyzed single-nucleotide polymorphisms (SNPs) from 291 accessions (Figure 4A), including 93 wild and 198 domesticated accessions, generated in a previous barley pangenome study [30]. Although this dataset was originally developed for characterizing structural variation revealed by pangenome in different accessions, we utilized the SNP dataset here to investigate population genomic signatures associated with domestication, including linkage disequilibrium and selective sweep detection. The phylogenetic analysis and principal component analysis (PCA) using whole-genome SNPs clearly separated all accessions into wild and domesticated groups (Figure 4B,C). The linkage disequilibrium (LD; measured as r2) dropped to half of its maximum value at approximately 401 kb for wild and 2.12 Mb for domesticated accessions (Figure 4D), comparable to wheat [43] but substantially larger than most cultivated crops, such as rice (20 kb for wild and 123 kb for cultivars) [44] and maize (22 kb for wild and 30 kb for cultivars) [45].

**Figure 5:**
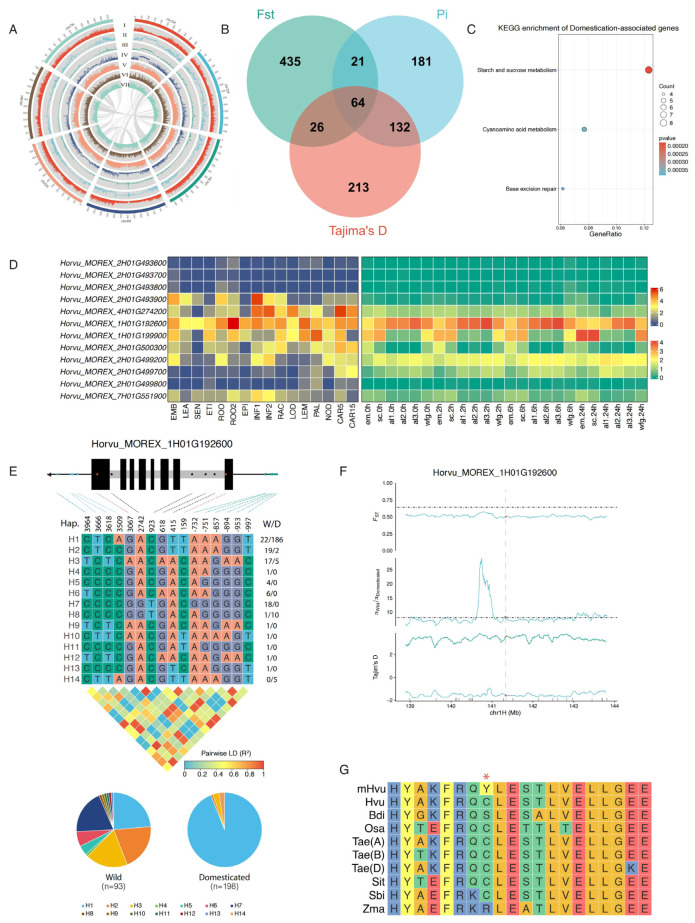
Identification and KEGG enrichment of candidate domestication-associated genes and selection analysis of a hexokinase-encoding gene Horvu_MOREX_1H01G192600. (**A**) Circos plot showing the density distributions of genomic features along barley chromosomes: SNPs (I), *F*_ST_ (II), π (III), and Tajima’s D (IV) in contiguous 100 kb windows and the intact LTR elements (V), Copia (VI), and Gypsy elements (VII) in contiguous 5 Mb windows. (**B**) The overlap of genes in the selective sweeps identified by *F*_ST_, π, and Tajima’s D methods with the top 1% values as the thresholds. (**C**) KEGG analysis of candidate domestication-associated genes. (**D**) Gene expression profiles of KEGG enriched genes in different developmental tissues [27] and seed tissues during germination [46]. The values of Transcripts Per Million reads (TPM) were transformed by log_2_(TPM + 1). EMB, 4 days after planting (dap) embryo; LEA, 17 dap leaf; SEN, 56 dap senescing leaf; ETI, 10 dap etiolated leaf grown in dark; EPI, 28 dap epidermal strips; ROO, 17 dap root; ROO2, 28 dap root; INF1, 30 dap inflorescences; INF2, 50 dap inflorescences; RAC, 35 dap inflorescence rachis; LOD, 42 dap lodicule; LEM, 42 dap lemma; PAL, 42 dap palea; NOD, the third stem internode (42 dap); CAR5, 5 days post-anthesis (dpa) caryopsis; CAR15, 15 dpa caryopsis; em, embryo; sc, scutellum; al1, one third of aleurone proximal to the embryo; al2, the central third of aleurone; al3, one third of aleurone distal to embryo; wfg; whole fixed grain; 0 h, 2 h, 6 h and 24 h represent the corresponding time after seed germination. (**E**) The haplotype and genotype frequency analysis of Horvu_MOREX_1H01G192600 during barley domestication. The SNPs in the upstream, CDS, intron, and downstream are marked in green, red, black, and blue colors, respectively, labeled with positions relative to the gene start position. The gene direction in the barley reference genome is indicated with a black arrow. (**F**) The *F*_ST_, π, and Tajima’s D values within the 5 Mb genomic region around Horvu_MOREX_1H01G192600. The dashed horizontal and vertical lines denote the genome-wide threshold values (top 1%) of selection signals (*F*_ST_: 0.64; π: 8.04) and the gene position of Horvu_MOREX_1H01G192600, respectively. The vertical lines in the *x*-axis indicate the genes within this region with Horvu_MOREX_1H01G192600 marked in red color. (**G**) The alignment of amino acids around the nonsynonymous mutation site of Horvu_MOREX_1H01G192600 among different grass species. Abbreviation: Osa, *Oryza sativa*; Sbi, *Sorghum bicolor*; Zma, *Zea mays*; Hvu, *Hordeum vulgare*; Tae, *Triticum aestivum*; Bdi, *Brachypodium distachyon*; Sit, *Setaria italica*. mHvu represents the mutated version relative to Horvu_MOREX_1H01G192600 in Morex.

Genome-wide population differentiation (*F*_ST_), nucleotide diversity (π) ratios, and Tajima’s D statistics were calculated between wild and domesticated populations (Figure 5A), and genomic regions showing top 1% values of highest *F*_ST_ values, π_wild_/π_domesticated_ ratios, or Tajima’s D difference values (ΔD = D_wild_ − D_domesticated_) were defined as selective sweeps. A total of 546, 398, and 435 genes overlapped with the selective sweeps identified by *F*_ST_, π, and Tajima’s D approaches, respectively. The 243 genes identified by at least two approaches were considered as candidate domestication-associated genes (Figure 5B and Table S8). The KEGG enrichments analysis showed that candidate domestication-associated genes were significantly enriched in starch and sucrose metabolism, cyanoamino acid metabolism, and base excision repair (Figure 5C and Table S9).

To further characterize the KEGG-enriched genes, their expression patterns were examined in different developmental [27] and germinating tissues [46] (Figure 5D). We then focused on a hexokinase-encoding gene Horvu_MOREX_1H01G192600, which exhibited high transcriptional levels in the examined tissues (Figure 5D). Hexokinases play important roles in glucose sensing and sugar metabolism and thereby influence plant growth and development [47]. A total of 16 SNPs were identified within Horvu_MOREX_1H01G192600 and its 1 kb flanking regions, including six upstream SNPs, one synonymous and one nonsynonymous SNPs in the exons, four intronic SNPs, and four downstream SNPs (Figure 5E and Table S10). These SNPs defined 14 haplotypes (H1-H14), with the frequency of H1 increasing from 23.7% (22/93) in the wild to 93.9% (186/198) in the domesticated population (Figure 5E). Furthermore, Horvu_MOREX_1H01G192600 was located within a selective sweep detected by both π and Tajima’s D statistic (Figure 5F). The amino acid corresponding to nonsynonymous mutation of Horvu_MOREX_1H01G192600 was not conserved among grass plant species (Figure 5G), and the frequency of the nonsynonymous mutation did not show substantial divergence between wild (20.4%; 19/93) and domesticated (2.5%; 5/198) populations (Figure 5E). However, the variant frequency of downstream SNP (c. A3509C) was decreased from wild (76.3%; 71/93) to domesticated populations (3.5%; 7/198) (Figure 5E), consistent with strong selection signals during domestication (Figure 5F).

**Figure 6:**
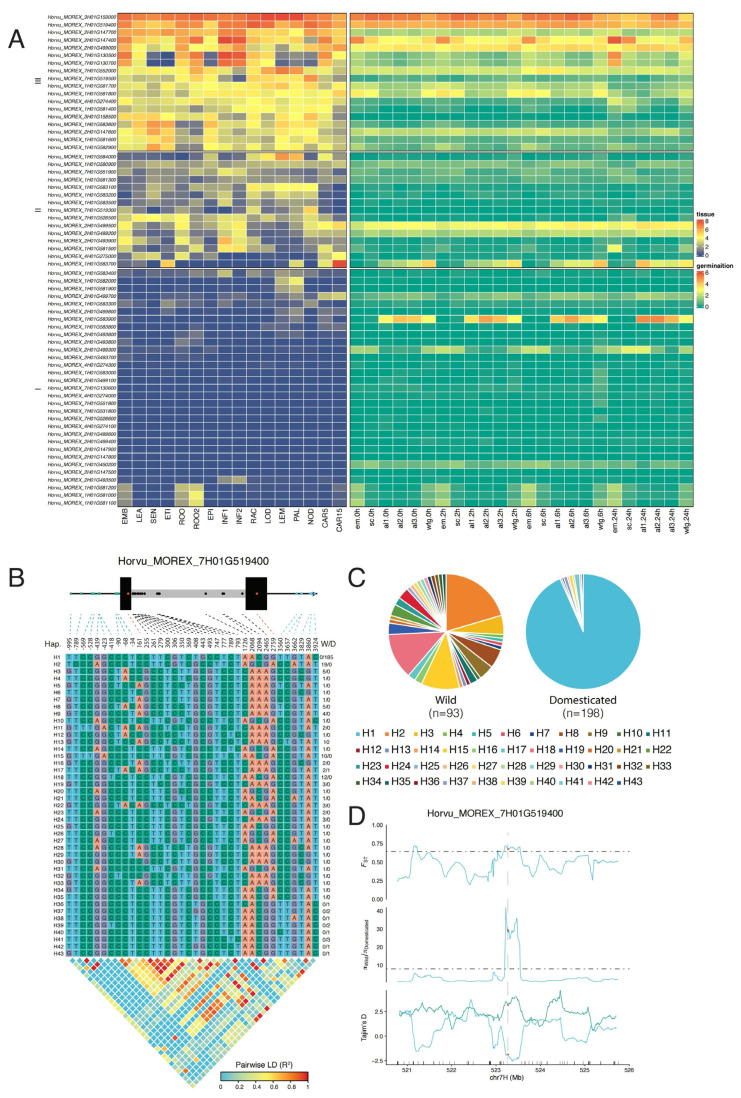
Transcriptional profiles of the domestication-associated genes simultaneously identified by three methods and selection analysis of a GTPase-encoding gene Horvu_MOREX_7H01G519400. (**A**) Gene expression profiles. The values of Transcripts Per Million reads (TPM) are transformed by log_2_(TPM + 1). (**B**,**C**) The genetic variations (**B**) and genotype frequency (**C**) analysis of Horvu_MOREX_7H01G519400 during barley domestication. The SNPs in the upstream, CDS, intron, and downstream are marked in green, red, black, and blue colors, respectively, labeled with positions relative to the gene start position. The gene direction in the barley reference genome is indicated with a black arrow. (**D**) The *F*_ST_, π, and Tajima’s D values within the 5 Mb genomic region around Horvu_MOREX_7H01G519400. The dashed horizontal and vertical lines denote the genome-wide threshold values (top 1%) of selection signals (*F*_ST_: 0.64; π: 8.04) and the gene position of Horvu_MOREX_7H01G519400, respectively. The vertical lines in the *x*-axis indicate the genes within this region with Horvu_MOREX_7H01G519400 marked in red color.

A total of 64 candidate domestication-associated genes were identified using three methods and clustered into three groups based on their transcriptional expressions, with group III showing high expression levels in different examined tissues (Figure 6A). Based on functional annotation, we further focused on a GTPase-encoding gene Horvu_MOREX_7H01G519400, as GTPases are crucial switches by binding GTP and GDP and regulate diverse cellular processes [48]. A total of 36 SNPs were identified within Horvu_MOREX_7H01G519400 and its 1 kb flanking regions, including ten upstream SNPs, two synonymous SNPs in the exons, 18 intronic SNPs, and six downstream SNPs (Figure 6B and Table S11). These SNPs defined 43 haplotypes (H1-H43), with the frequency of H1 increased from 0% (0/93) in the wild populations to 93.4% (185/198) in the domesticated populations (Figure 6B,C). Notably, Horvu_MOREX_7H01G519400 was located in a selective sweep consistently identified using all three methods (Figure 6D). The three downstream SNPs (c. T3560C, T3657C, and C3924T) exhibited strong linkage disequilibrium (Figure 6B), and their variant frequencies decreased from wild (89.3%, 83/93; 98.9%, 92/93; 98.9%, 92/93) to domesticated populations (1.0%, 2/198; 0.5%, 1/198; 0.5%, 1/198), consistent with strong selection signals during domestication (Figure 6D). These population genomic analyses identified candidate regions potentially shaped by domestication selection. However, whether domestication-associated processes are accompanied by changes beyond DNA sequence variation, such as alterations in higher-order genome organization, remains unclear. We therefore compared Hi-C datasets from a domesticated cultivar and two wild barley accessions to characterize differences in chromatin architecture.

### 2.5. Comparative Hi-C Analyses Reveal Divergent A/B Compartment Organization Among Three Barley Accessions

**Figure 7:**
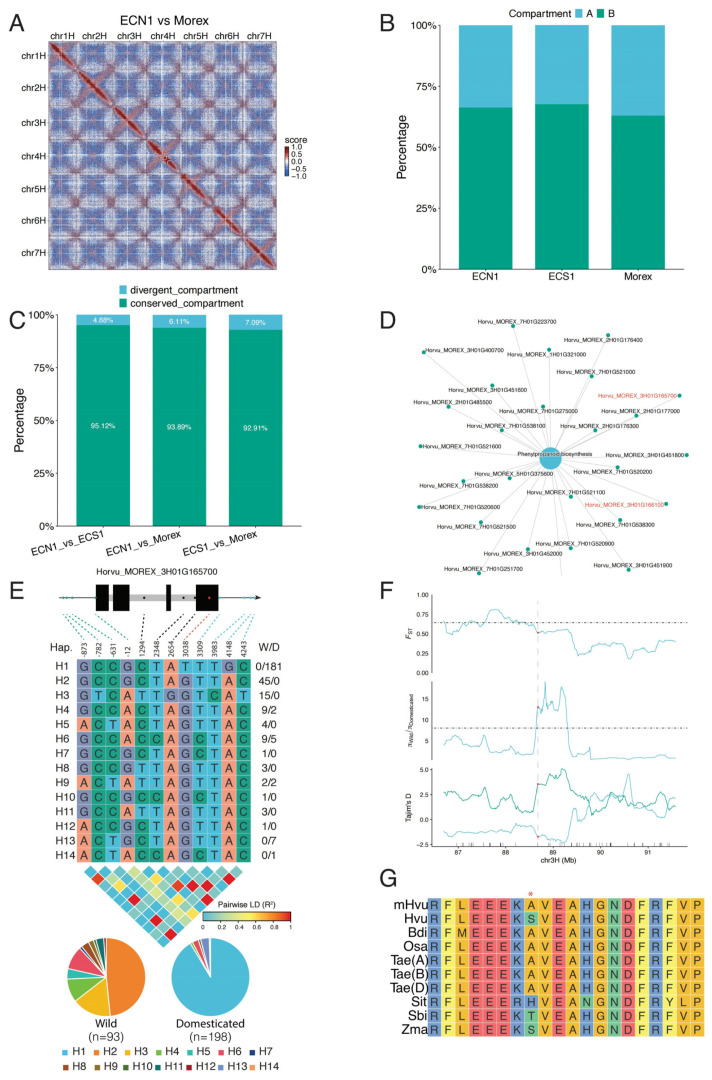
Comparison of A/B compartment organization among three barley accessions and selection analysis of a cinnamate-4-hydroxylase-encoding gene Horvu_MOREX_3H01G165700. (**A**) The genome-wide log_2_-transformed ratio of normalized and corrected Hi-C contact matrices between ECN1 and Morex in contiguous 1 Mb windows at 70 kb resolution. The red and blue indicate contact enrichment in ECN1 and Morex, respectively. (**B**) The proportion of A/B compartments in the three barley genomes at 70 kb resolution. (**C**) Comparison of conserved and divergent compartment regions among the three barley genomes at 70 kb resolution. (**D**) The genes with B-to-A compartment transitions in comparison of ECN1 vs. Morex are enriched in phenylpropanoid biosynthesis. Red colors mark the domestication-associated genes. (**E**) The haplotype and genotype frequency analysis of Horvu_MOREX_3H01G165700 during barley domestication. The SNPs in the upstream, CDS, intron, and downstream are marked in green, red, black, and blue colors, respectively, labeled with positions relative to the gene start position. The gene direction in the barley reference genome is indicated with a black arrow. (**F**) The *F*_ST_, π, and Tajima’s D values within the 5 Mb genomic region around the Horvu_MOREX_3H01G165700. The dashed horizontal and vertical lines denote the genome-wide threshold values (top 1%) of selection signals (*F*_ST_: 0.64; π: 8.04) and the gene position of Horvu_MOREX_3H01G165700, respectively. The vertical lines in the *x*-axis indicate the genes within this region with Horvu_MOREX_3H01G165700 marked in red color. (**G**) The alignment of amino acids around the nonsynonymous mutation site of Horvu_MOREX_3H01G165700 among different grass species. The red stars denote the nonsynonymous mutations. Abbreviation: Osa, *Oryza sativa*; Sbi, *Sorghum bicolor*; Zma, *Zea mays*; Hvu, *Hordeum vulgare*; Tae, *Triticum aestivum*; Bdi, *Brachypodium distachyon*; Sit, *Setaria italica*. mHvu represents the mutated version relative to Horvu_MOREX_3H01G165700 in Morex.

Dynamic changes in 3D genome architecture are associated with gene regulation, plant growth, development, and environmental responses [9,10]. To investigate the variation in chromatin organization among barley accessions, we obtained the reference genome assemblies [30,32] and seedling leaf Hi-C datasets [27,32] of the domesticated cultivar Morex and two wild barley accessions (ECN1 and ECS1). The wild barley accession ECN1 was from the north slope of evolution canyon with a cooler climate and higher relative humidity, whereas the wild barley accession ECS1 was from the south slope with higher daily temperatures [32]. Comparable Hi-C contact proportion of intra- (71.32–76.22%) and inter-chromosome contacts (23.78–28.68%) were observed for the three accessions (Table S12). We observed strong contact signals in the diagonals as expected and relatively weak anti-diagonal signals in the intrachromosomal Hi-C contact matrices of all three genomes (Figure S21), consistent with previous reported Rabl-type configurations in barley [27]. Furthermore, comparative analysis of Hi-C contact signals revealed differences in chromatin contact patterns among the three accessions, with ECN1 and ECS1 showing higher short-range chromatin contact frequencies than Morex (Figure 7A and Figure S22A).

Plant chromatin is broadly partitioned into euchromatic A compartments and heterochromatic B compartments [9,10]. We annotated the compartment regions in the three barley genomes and found that Morex contained a higher proportion of A compartment than ECS1 or ECN1 (Figure 7B). We observed approximately 6.11% and 7.09% divergent compartment regions in the comparisons of ECN1 vs. Morex and ECS1 vs. Morex, respectively, and around 4.88% between ECN1 and ECS1 (Figure 7C). These results indicated that compartment organization differed among the three barley accessions, with larger differences observed between Morex and each wild accession than between the two wild accessions. In total, 1445 and 2037 genes exhibited B-to-A compartment transitions in the comparisons of ECN1 vs. Morex (Table S13) and ECS1 vs. Morex (Table S14), respectively, whereas only 798 and 660 genes showed A-to-B compartment transitions in the corresponding comparisons. The genes with B-to-A compartment transitions in ECN1 or ECS1 vs. Morex were enriched in the phenylpropanoid biosynthesis pathway (Figure 7D and Figure S22B; Tables S15 and S16). The phenylpropanoid metabolism plays crucial roles in plant defense and stress tolerance in plant [49], raising the possibility that artificial selection on genes in this pathway contributed to enhanced environmental adaptation during barley domestication. To further evaluate this possibility, we overlapped phenylpropanoid biosynthesis-enriched genes in the comparisons of ECN1 vs. Morex and ECS1 vs. Morex with candidate domestication-associated genes and identified two shared genes (a cinnamate-4-hydroxylase-encoding gene Horvu_MOREX_3H01G165700 and a peroxidase-encoding gene Horvu_MOREX_3H01G166100) (Figure 7D and Figure S22B).

A total of 12 SNPs were observed within Horvu_MOREX_3H01G165700 and its the 1 kb flanking regions, including four upstream SNPs, one nonsynonymous SNP in the exons, three intronic SNPs, and four downstream SNPs (Figure 7E and Table S17). These SNPs defined 14 haplotypes (H1-H14), and the frequency of H1 increased from 0% (0/93) in the wild to 91.4% (181/198) in the domesticated population (Figure 7E). Furthermore Horvu_MOREX_3H01G165700 was located within a selective sweep detected based on π and Tajima’s D statics (Figure 7F). The nonsynonymous (c. T3038G) and the downstream SNPs (c. G4148A) showed strong linkage disequilibrium (Figure 7E), and their variant frequencies decreased from wild (100%, 93/93) to domesticated populations (8.6%, 17/198), consistent with selection signals associated with domestication (Figure 7F). However, the amino acid corresponding to nonsynonymous mutation of Horvu_MOREX_3H01G165700 was not conserved among grass plant species (Figure 7G), suggesting the downstream SNPs (c. G4148A) might be the site under artificial selection. Similarly, 25 SNPs in the candidate domestication-associated gene Horvu_MOREX_3H01G166100 defined 19 haplotypes (H1-H19), and the frequency of H1 increased from 0% (0/93) in the wild to 91.4% (181/198) in the domesticated population (Figure S22C and Table S18). The three downstream SNPs (c. C1743T, C1560G, and T1439C) of Horvu_MOREX_3H01G166100 showed strong linkage disequilibrium (Figure S22C), and variant mutations deceased from wild (100%, 93/93; 98.9%, 92/93; 98.9%, 92/93) to domesticated population (8.1%, 16/198; 8.1%, 16/198; 7.6%, 15/198), consistent with selection signals associated with domestication (Figure S22D). These results revealed the dynamic A/B compartment reorganization among the three barley accessions. Because A/B compartment transitions represent only one level of chromatin organization, we further compared TAD organization among the three barley genomes.

### 2.6. Comparative TAD Analyses Reveal Divergent Chromatin Domain Organization Among Three Barley Accessions

**Figure 8:**
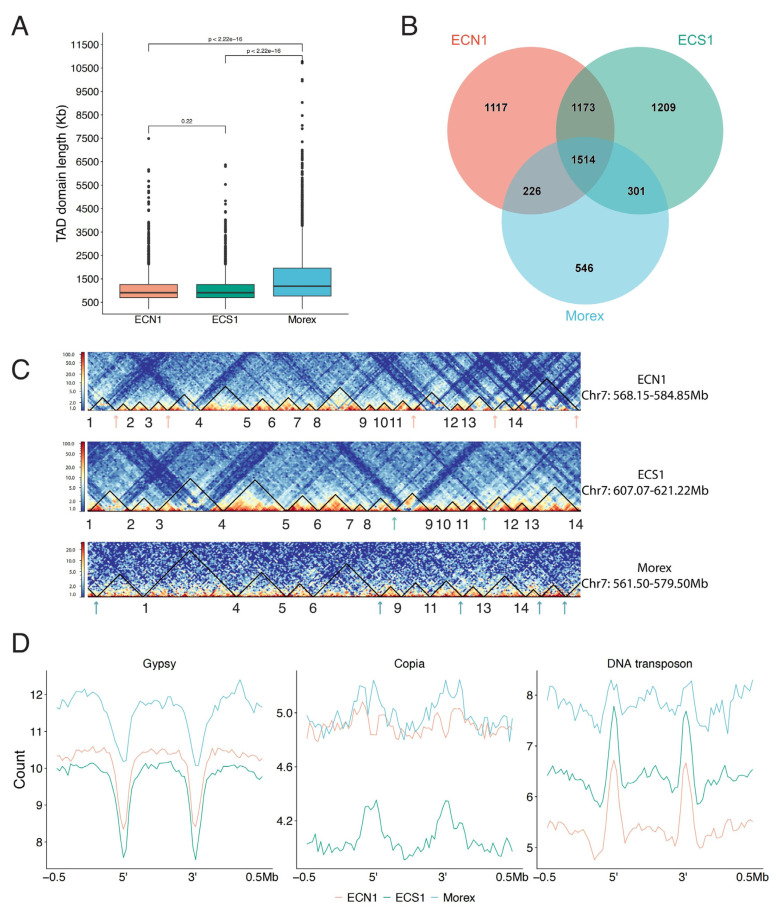
Comparative analysis of topologically associating domains (TADs) and transposable element distributions around TADs among three barley accessions. Boxplots of TAD domain lengths (**A**) and conservation of TAD boundaries (**B**) at 70 kb resolution. *p* values of Wilcoxon rank-sum test are indicated in (**A**). (**C**) The TAD structures in the syntenic blocks on chromosome 7 among the three genomes at 70 kb resolution. The log-transformed Hi-C contact matrices are shown. The conserved TAD boundaries among the three genomes are denoted by black numbers. The colored arrows indicate accession-specific TAD boundaries. (**D**) Distributions of transposable elements around TAD domains among the three barley genomes using 20 kb windows.

To characterize TAD organization of TADs among the three accessions, we annotated their TAD domains and identified 4162, 4355, and 2644 TAD domains in ECN1, ECS1, and Morex, covering 99.40%, 99.28%, and 99.37% of the respective genomes (Table S19). Morex exhibited significantly larger TAD size than ECN1 or ECS1 (Figure 8A). To assess the conservation and variation of the TAD boundaries among the three accessions, we clustered TAD boundaries and found that 1514 boundary clusters were shared across the three genomes (Figure 8B and Table S20). Notably, a substantial proportion of accession-specific TAD boundary clusters were observed, with 27.7% (1117/4030) in ECN1, 28.8% (1209/4197) in ECS1, and 21.1% (546/2587) in Morex (Figure 8B), indicating substantial variation in TAD boundary among the three accessions. For example, within the syntenic regions on chromosome 7, we identified 14 conserved TAD boundaries between ECN1 and ECS1 along with five ECN1-specific and two ECS1-specific boundaries. In contrast, only eight of the 14 boundaries were retained in Morex, which also contained five Morex-specific boundaries (Figure 8C). Because TE dynamics have been suggested to be associated with TAD boundary reorganization in plants [50], we investigated the TE density around TAD boundaries in the three accessions and observed the lower Gypsy density but higher Copia and DNA transposon densities at the TAD boundaries (Figure 8D), suggesting that Copia retrotransposons and DNA transposons may be associated with higher-order chromatin organization in barley.

Gene family clustering analysis revealed a high degree of conservation among the three accessions, with 21,311 gene families shared, accounting for 81.5% of total gene families in ECN1, 80.9% in ECS1, and 86.7% in Morex, respectively (Figure S23). These results demonstrated that although gene count and predicted molecular functions are highly conserved among the three barley accessions, higher-order regulatory features, particularly TAD organization, show substantial variation.

## 3. Discussion

Barley, which exhibits strong adaptation to diverse environments, ranks as the fourth most important cereal crop worldwide in terms of harvested area and production, and serves as a major resource for livestock feed and brewing industries [26]. The increasing frequency of extreme heat and drought events has imposed substantial negative impacts on barley production and global beer supply [51]. The long-term artificial selection of barley has significantly reduced the genetic diversity of modern cultivars and promoted genetic homogeneity [52]. Therefore, a comprehensive understanding of barley genomic evolution and domestication is essential to develop environmentally resilient and high-performing cultivars. Our integrated analyses provide complementary insights into barley genome evolution from four interconnected perspectives: genome evolution, epigenetic regulation, domestication-associated selection, and high-order chromatin organization.

Our study indicates that extensive proliferation of TEs represents an important contributor to barley genome expansion (Figure 2), particularly Copia and Gypsy LTR retrotransposons, a feature commonly observed in plant species with large genomes [3]. A recent segmental duplication (Ks ~ 0.2) observed in barley (Figure 1E) is consistent with previously reported TE-driven gene duplication in Triticeae species [37] and might have provided additional genetic materials for evolutionary diversification. The enrichment of barley-specific genes in the mismatch repair pathway may reflect lineage-specific evolutionary processes and requires further investigation. The absence of WGD or WGT after divergence from sorghum (Figure 1F,G) as well as high collinearity with wheat (Figure S8) establishes barley as an informative model crop for genomic investigation within the Triticeae.

Long-term domestication has led to a suite of traits that distinguish cultivated barley from its wild progenitors, such as row type of spike and grain dispersal [26]. Although barley was first domesticated in the Fertile Crescent before spreading to other regions [18], the mosaic genomic ancestry of barley populations [18], which results in high haplotype differentiation among geographically distinct barley populations, poses challenges for mapping domesticated genes. Here, population genomic analyses identified a set of candidate domestication-associated genes, which were enriched in sucrose metabolism-related pathways. These candidates provide potential targets for future functional studies aimed at understanding the genetic basis of barley domestication and improvement. It should be emphasized that candidate domestication-associated genes were identified without explicitly accounting for the geographic structure of wild and domesticated populations, largely due to limited resequencing sample size. With the accumulation of geographically representative resequencing datasets, integrating geographic population structure will enable more precise dissection of domestication signals and reduce confounding effects of population differentiation. In addition, it should be noted that no phenotypic validations were employed in domestication-associated gene mining, future genetic and phenotypic validation, such as gene editing and transgenic analyses, will be necessary to determine the contribution of these candidate genes to barley domestication-associated traits. Besides genes, TE insertion polymorphisms (TIPs) also contribute to plant diversity [5] and could undergo artificial selection by affecting nearby gene expressions during crop domestication [4]. It will be of interest in future studies to investigate the potential contributions of TIPs to barley genome evolution and domestication. Recent barley pangenome study [28] have revealed extensive structural variations among wild and domesticated accessions. To integrate these structural variations with domestication-associated signals identified in our study, we performed a comparative analysis of their overlap. Because our study was based on the Morex reference genome v2 [30], whereas the reported pangenome structural variation hotspots were annotated using the Morex reference genome v3 [28], we first converted the coordinates of the reported hotspot regions from v3 to v2. After filtering converted regions with a mapping ratio > 0.7, 86 out of 169 CNV hotspot regions, containing 543 genes, were retained for downstream comparison (Table S21). No direct overlap was observed between these genes and our candidate domestication-associated genes. The absence of overlap may be partly attributed to differences between the two reference genome assemblies and the challenges in accurately transferring structural variation regions, particularly CNV-associated regions, between genome versions. Future studies based on improved pangenome references and population-scale structural variation datasets will facilitate a more comprehensive understanding of the contribution of structural variation to barley domestication.

Strong positive correlations of TEs and methylation levels of mCG or mCHG contexts were observed across plant species at genome-wide and chromosome scales (Figure 3A,C and Figure S20), suggesting a close association between DNA methylation on TE transposition, consistent with previous reports [2,6]. It should be emphasized the current methylome analysis represents a comparative characterization of a representative wild barley accession, and future generation of methylome datasets from diverse wild and domesticated barley accessions will be necessary to investigate epigenetic changes during barley domestication.

The 3D architecture dynamics of plant genomes are associated with gene regulations [11] and environmental responses [14] and also are involved in crop domestication [13]. To compare the compartment transitions in the same genomic coordinate system, we mapped the two wild barley reads to the domesticated Morex reference genome, whereas this strategy might introduce reference mapping bias. For example, the genomic divergence of ECN1 or ECS1 from Morex might have lower coverage or mapping quality, artifactually reducing Hi-C signals and potentially inflating apparent A/B compartment differences. High conservation of gene content and substantial divergence of TAD boundaries were observed among the three barley accessions (Figure 8B,C and Figure S23). In animals, the TAD boundaries are established by CTCF binding sites, which facilitate the recruitment of CTCF proteins and cohesion [11]. However, plants lack CTCF proteins, and the enrichment of specific TE classes at TAD boundaries suggests a potential association between TEs and TAD organization in barley (Figure 8D). To investigate the characteristics of candidate domestication-associated genes, we analyzed the genomic distribution of these genes relative to A/B compartments and TAD boundaries. Among these genes, 114 were located within A compartments and 129 within B compartments, while 19 genes overlapped with TAD boundary regions of Morex (Table S8), suggesting no preferential enrichment in either compartment. It should be noted that our chromatin organization analysis only involved one domesticated cultivar and two wild barley accessions. Future analyses using larger collections of wild and domesticated accessions will be required to eliminate the individual-specific variations and identify the domestication-associated chromatin changes in the future.

Together, our comparative genomic analyses indicate that lineage-specific TE proliferation was likely an important contributor to barley genome expansion, while comparative methylome analyses reveal characteristic DNA methylation landscapes associated with TE-rich genomes. Population genomic analyses further identified candidate genomic regions potentially shaped by domestication-associated selection. Comparative Hi-C analyses of Morex, ECN1, and ECS1 uncover differences in higher-order chromatin organization among these three barley accessions, suggesting that chromatin organization may diverge despite conservation of gene content. Although the biological significance of these genomic and chromatin differences requires further investigation using broader barley populations and functional validation, the integration of comparative genomics, epigenomics, population genomics, and chromatin analyses provides a comprehensive framework for understanding barley genome evolution and domestication.

## 4. Materials and Methods

### 4.1. Genomic, Resequencing, and RNA-Seq Data Resources

The reference genomes of *Hordeum vulgare* [30], *Arabidopsis thaliana* [53], and four taxa in the Poaceae (grass) clade, including *Sorghum bicolor* [54], *Zea mays* [55], *Oryza sativa* [56], and *Triticum aestivum* [57], were downloaded from public repositories or specific websites (Table S1). Only chromosome-level assemblies were considered for subsequent analysis (TE annotation, methylation level detection, and synteny analysis), while scaffold sequences were excluded.

The VCF file containing SNP variations of 291 diverse barley (*Hordeum vulgare*) varieties, including 93 wild and 198 domesticated accessions, were downloaded from a previously published study [30].

The raw RNA-Seq data of different developmental tissues [27] and different tissues of germinating seeds [46] were derived and mapped to reference genome [30] of barley to calculate gene expressions following the previous described methods [58].

### 4.2. Population Genetics Analysis and SNP Annotation

The geographic distribution of downloaded resequencing barley varieties was shown using R package maps (version 3.4.3) and ggplot2 (version 4.0.0).

The downloaded SNPs with minor allele frequency lower than 5% and missing rate larger than 10% in the population were discarded using VCFtools [59] (version 0.1.17). Then, the package beagle (version 5.4) with the default parameters were employed for imputation of missing data. A total of 33,073,566 post-filtering SNPs were used to calculate the linkage disequilibrium (LD) decay distance within wild and domesticated groups using PopLDdecay and annotated based on the barley reference genome [30] using ANNOVAR (version 2019-10-24).

The VCFtools (version 0.1.17) was employed with 100–10 kb sliding windows to calculate the pairwise genetic differentiation index *F*_ST_ values between wild and domesticated populations, and π and Tajima’s D values within wild and domesticated populations. The ratio of diversity (π_wild_/π_domesticated_) and difference in Tajima’s D between wild and domesticated populations were calculated. For each method, the genomic regions with top 1% highest *F*_ST_ values, highest π_wild_/π_domesticated_ ratios, or highest Tajima’s D difference values (ΔD = D_wild_ − D_domesticated_) were determined as selective sweeps, and the genes that overlapped with selective sweeps identified in at least two methods were identified as candidate domesticated genes.

The filtered SNPs were further filtered by PLINK [60] (version 1.90) using the following the parameter: --indep-pairwise 50 10 0.2. The filtered SNPs were used to carry out phylogenetic tree and principal component analysis (PCA) of resequencing accessions. The neighbor-joining phylogeny tree was constructed using PHYLIP software (version 3.697) and shown with iTOL (https://itol.embl.de/). The PCA analysis was performed using PLINK (version 1.90) with the following parameter: --pca set as 291. The first and second eigenvectors were plotted.

### 4.3. Enrichment Analysis, Gene Expression Illustration, and Haplotype Analysis

The R package clusterProfiler (version 4.8.2) was employed to perform the KEGG and GO enrichment analysis, and the results were shown using R package ggplot2 (version 3.5.2). The R package ComplexHeatmap (version 2.10.0) was used to generate the heatmaps of gene expression profiles based on the log_2_(TPM +1) transformation.

The SNPs within and in the 1 kb flanking regions of candidate genes were extracted to perform haplotype analysis. PLINK [60] (version 1.90) was employed to calculate the pairwise linkage disequilibrium (R^2^) of SNPs, and the results were displayed with R package LDheatmap (version 1.0-6).

### 4.4. Evolution Analysis

The longest translation form of each gene from five taxa (barley, sorghum, rice, maize, wheat) in the grass clade and the outgroup Arabidopsis, downloaded from public repositories or specific websites, were used to perform comparative genomics analysis. Gene family clustering was conducted using OrthoFinder [61] (version 2.5.4). The orthogroups containing three genes in wheat and only one gene in other plant species were filtered, and we randomly discarded two wheat genes in each orthogroups to generate the super genes for construction of maximum-likelihood (ML) species phylogenetic tree using IQ-Tree (version 1.6.12) with the following parameters: -m MFP -bb 1000. The gene family expansion and contraction along plant phylogenetic tree was estimated using CAFE5, based on the divergence time of the six plant species obtained from TimeTree (https://timetree.org/).

The genome-wide syntenic analysis within or among the six plant species were carried out as described previously [58]. The synteny illustrations among different plant species were generated using python version JCVI (https://github.com/tanghaibao/jcvi, accessed on 30 March 2024) of MCScan. The syntenic blocks containing >200 genes among different plant species were shown in yellow colors. The duplicated genes within a plant species were classified into whole genome/segmental, tandem, proximal or dispersed duplications using duplicate_gene_classifier [62] with default parameters.

The reciprocal best hit orthologs between different plant species were determined using WGD [63], and their protein sequences were aligned and then transformed into nucleotide sequences using ParaAT. The KaKs_Calculator 2.0 was employed to estimate the synonymous nucleotide substitution per synonymous site (Ks) using the Yang–Nielsen model [64]. The syntenic gene pairs in the syntenic blocks within plant species were used to calculate Ks values as described above. The R package ggridges (version 0.5.6) was employed to display the density distribution of Ks.

### 4.5. Transposable Element (TE) Annotation and LTR Insertion Time Analysis

The TE annotation of a plant genome was first conducted using EDTA [65] (version 2.0.1) with the following parameters: --step all --sensitive 0 --anno 1. Since a proportion of long terminal repeat retrotransposons (LTRs) in the TE library generated in the original EDTA pipeline could not be classified into a specific superfamily, DeepTE was then used to further classify these unknown LTR. Finally, the improved non-redundant TE library was used as input to produce TE annotation in the plant genome with the following parameters: --step anno --overwrite 1 --anno 1. The LTR insertion time was estimated using full-length LTRs with neutral mutation rate of 1.3 × 10^−8^ per bp per year.

### 4.6. Phylogenetic Tree of Full-Length LTR of Barley and Rice

The full-length LTRs of barley and rice were extracted using BEDtools (version 2.31.1) according to gff3 files generated in EDTA pipeline and classified at clade levels using TEsorter [66] with rexdb-plant repeat database [67]. The RT domain larger than 200 amino acids of full-length Copia LTR of barley and rice were extracted and aligned using MUSCLE (version 3.8.31). The phylogenetic tree of Copia elements of barley and rice was constructed using IQ-TREE (version 1.6.12) and shown with iTOL (https://itol.embl.de/). The same methods were employed for phylogenetic tree of RT domain (larger than 150 amino acids) of full-length Gypsy LTR of barley and rice. The insertion time of full-length LTR was displayed using the R package ggridges (version 0.5.6).

### 4.7. Circos Plots

The density distributions of SNPs, *F*_ST_, π, and Tajima’s D in contiguous 100 kb windows and the density distributions of the intact LTR elements as well as Copia and Gypsy elements in contiguous 5 Mb windows across barley genome were displayed using Circos (version 0.69-8).

### 4.8. WGBS Analysis

Given that the whole-genome bisulfite sequencing raw data of barley [31] was from wild accession Rossa166, we also downloaded the resequencing data of Rossa166 to construct a pseudo-genome for methylation level analysis as previously described [31], eliminating the interference of nucleotide variations. The whole-genome bisulfite sequencing raw data of barley accession Rossa166 was filtered using fastp (version 0.12.4) with the default parameters. The high-quality clean reads were mapped to the pseudo reference genome using Bismark (version 0.24.2) with default parameters. The methylation level for each cysteine site was calculated using BatMeth2 [68] with the following command: BatMeth2 calmeth -Q 20 --remove_dup --coverage 4 -nC 1. The methylation level within gene or TE and flanking regions were conducted using BatMeth2 [68] with the following command: BatMeth2 methyGff --coverage 4 -nC 1 --distance 2000 --step 0.01. The visualization of methylation level was conducted using ggplot2 (version 3.5.2).

The whole-genome bisulfite sequencing raw data of Arabidopsis [69], sorghum [70], rice [71], maize [72], and wheat [73] were from the same varieties were used for reference genome assemblies. The methylation levels of these plant species were conducted as described above without construction of pseudo-genomes.

### 4.9. A/B Compartment Identification

The Hi-C sequencing data of barley varieties Morex [27], EC_N1, and EC_S1 [32] were downloaded from previous studies. First, the Hi-C sequencing data were filtered using fastp (version 0.12.4) with the default parameters and mapped to the reference genome of Morex [30] using bwa (version 0.7.17) with the following parameters: bwa mem -A1 -B4 -E50 -L0. The Hi-C contact matrix was generated using hicBuildMatrix [74] (version 3.7.5) with the following parameters: --binSize 10000 --minDistance 500 --maxLibraryInsertSize 1500 --restrictionSequence AAGCTT --danglingSequence AGCT. Next, hicNormalize [74] (version 3.7.5) was employed to normalize contact matrices among three varieties with the following parameters: --normalize smallest. Then, the matrices resolutions were zoomified using Cooler (version 0.10.2) with iterative correction and eigenvector decomposition (ICE) method. Finally, the 70 kb resolutions for which 80% of loci showed at least 1000 contacts were selected to perform compartment analysis using R package Calder [75] with gene density as compartment A/B classification standard.

Hi-C contact matrices in chromosome-levels were displayed using plotMatrix from R package HiContacts (version 1.6.0) at 1 Mb resolution.

### 4.10. TAD Boundary Identification

The Hi-C reads of three barley varieties were processed as described in the compartment A/B identification, with the exception that they were mapped to their own reference genomes. The 70 kb resolution of contact matrices was used to detect the TAD boundaries using hicFindTADs [74] (version 3.7.5) with the default parameters. The conserved and specific TAD boundaries among the three varieties were identified using Tcbf [76]. The Hi-C matrices and TAD domains were displayed using pyGenomeTracks (version 3.9). The TE counts around TAD boundaries were calculated using Deeptools (version 3.5.5) and displayed using ggplot2 (version 4.0.0).

### 4.11. Statistics

Statistical analyses were performed in R (version 4.4.0). Pearson’s correlation and corresponding statistical significance analyses were assessed using the R package rstatix (version 0.7.2). Differences between groups were evaluated using the Wilcoxon rank-sum test. All statistical tests were two-sided unless otherwise specified. Exact *p* values are provided in the figures and/or figure legends.

## 5. Conclusions

In summary, our study integrates comparative genomics, epigenomics, population genomics and chromatin architecture analyses to investigate barley genome evolution and domestication from complementary perspectives. Comparative analyses identify lineage-specific genomic characteristics associated with barley genome evolution, while population genomic analyses reveal candidate genomic regions potentially shaped by domestication. Comparative Hi-C analyses further uncover differences in higher-order chromatin organization among three representative barley accessions. Although these findings remain largely correlative and require further validation using broader barley populations and functional studies, the integration of complementary multi-omics datasets provides a valuable framework for future investigations into the molecular mechanisms underlying barley evolution, domestication, and crop improvement.

## Data Availability

The reference genome assembly of *Hordeum vulgare* (cv. Morex) [30] is publicly available via barley pangenome repository at https://barley-pangenome.ipk-gatersleben.de (accessed on 30 January 2025). The genome assemblies of two wild barley accessions (ECN1 and ECS1) [32] have been deposited in the NCBI database under BioProject accession PRJNA947680. The reference genomes of *Arabidopsis thaliana*, *Sorghum bicolor*, *Zea mays*, *Oryza sativa*, and *Triticum aestivum* were obtained from Phytozome (https://phytozome-next.jgi.doe.gov/, accessed on accessed on 30 January 2025). The whole-genome bisulfite sequencing (WGBS) data of Arabidopsis (SRR13650156 and SRR13650157), maize (SRR12463972 and SRR12463973), barley (SRR13345339), and wheat (SRP133674) are accessible through Sequence Read Archive (SRA, https://www.ncbi.nlm.nih.gov/sra, accessed on 30 January 2025). The WGBS data of sorghum (CRR145305) and rice (CRR190552) are available in the Genome Sequence Archive (GSA, https://ngdc.cncb.ac.cn/gsa/, accessed on 30 January 2025). The Hi-C data of Morex (PRJEB14169), ECN1 (PRJNA748178) and ECS1 (PRJNA748178) have been deposited in the NCBI database. The SNP VCF file [30] of diverse barley accessions analyzed in this study was downloaded from https://doi.ipk-gatersleben.de/DOI/c4d433dc-bf7c-4ad9-9368-69bb77837ca5/1b898e9a-11bf-4eb6-b165-e1fa2167f0ec/0 (accessed on 30 January 2025). The RNA-Seq data of different developmental tissues [27] and different tissues of germinating seeds [46] of barley are available in NCBI database under PRJEB14349 and PRJNA378132, respectively. The datasets supporting the conclusions of this article are available in the Figshare repository (10.6084/m9.figshare.31563343) or included in the article and its Supplementary Materials. Additional materials and resources are available from the corresponding authors upon reasonable request.

## Supplementary Materials

The following supporting information can be downloaded at: https://www.mdpi.com/article/10.3390/plants15152341/s1, Figure S1: GO enrichment of barley-specific genes; Figure S2: Estimation of plant divergence (A) and whole-genome duplication (B) based on Ks distributions; Figure S3: Genomic syntenic analysis between barley (Hvu) and Arabidopsis (Ath); Figure S4: Genomic syntenic analysis between barley (Hvu) and rice (Osa) or sorghum (Sbi); Figure S5: Genomic syntenic analysis between barley (Hvu) and maize (Zma) or wheat (Tae); Figure S6: Dot plot analysis (A) and syntenic depth analysis (B) of genome-wide syntenic blocks between barley (Hvu) and rice (Osa); Figure S7: Dot plot analysis (A) and syntenic depth analysis (B) of genome-wide syntenic blocks between barley (Hvu) and maize (Zma); Figure S8: Dot analysis (A,C,E,G) and syntenic depth analysis (B,D,F,H) of genome-wide syntenic blocks between barley (Hvu) and wheat (Tae), wheat sub-genome A (TaeA), wheat sub-genome B (TaeB) or wheat sub-genome D (TaeD); Figure S9: Estimation of insertion times of Copia and Gypsy transposons in different plant species; Figure S10: DNA methylation levels in three sequence contexts in different plant genomes; Figure S11: Distributions of genes, LTRs, and DNA methylation levels of mCG, mCHG, and mCHH along chromosomes of different plant species; Figure S12: Distributions of genes, LTRs, and DNA methylation levels of mCG, mCHG, and mCHH along different chromosomes of Arabidopsis (Ath); Figure S13: Distributions of genes, LTRs, and DNA methylation levels of mCG, mCHG, and mCHH along different chromosomes of rice (Osa); Figure S14: Distributions of genes, LTRs, and DNA methylation levels of mCG, mCHG, and mCHH along different chromosomes of sorghum (Sbi); Figure S15: Distributions of genes, LTRs, and DNA methylation levels of mCG, mCHG, and mCHH along different chromosomes of maize (Zma); Figure S16: Distributions of genes, LTRs, and DNA methylation levels of mCG, mCHG, and mCHH along different chromosomes of barley (Hvu); Figure S17: Distributions of genes, LTRs, and DNA methylation levels of mCG, mCHG, and mCHH along different chromosomes of wheat sub-genome A (TaeA); Figure S18: Distributions of genes, LTRs, and DNA methylation levels of mCG, mCHG, and mCHH along different chromosomes of wheat sub-genome B (TaeB); Figure S19: Distributions of genes, LTRs, and DNA methylation levels of mCG, mCHG, and mCHH along different chromosomes of wheat sub-genome D (TaeD); Figure S20: The correlation between methylation levels of mCG, mCHG, and mCHH and gene (left panel of each species) or TE density (right panel of each species) using 1Mb sliding windows with 200 kb steps among different plant species; Figure S21: Genome-wide Hi-C contact maps of wild barley (ECN1 and ECS1) and Morex in contiguous 1 Mb windows at the resolution of 70 kb; Figure S22: A/B compartment reorganization during barley domestication and selection analysis of a peroxidase-encoding gene Horvu_MOREX_3H01G166100; Figure S23: Gene family clustering analysis among the genomes of wild barley (ECN1 and ECS1) and Morex; Table S1: The public resources used in this study; Table S2: Gene family clustering overall statistics; Table S3: Gene family clustering statistics of each species; Table S4: KEGG enrichment of barley-specific genes; Table S5: GO enrichment of barley-specific genes; Table S6: Gene duplication classifications of each plant species; Table S7: Statistics of transposable elements in different plant species in this study; Table S8: Annotations and gene expressions of candidate domestication-associated genes; Table S9: KEGG enrichment of candidate domestication-associated genes in barley; Table S10: The annotation of SNP variations in Horvu_MOREX_1H01G192600; Table S11: The annotation of SNP variations in Horvu_MOREX_7H01G519400; Table S12. Summary of Hi-C sequencing depth and contact matrix quality statistics; Table S13: The annotation of genes with compartment transitions in the comparison of ECN1 vs. Morex; Table S14: The annotation of genes with compartment transitions in the comparison of ECS1 vs. Morex; Table S15: KEGG enrichment of genes with B-to-A compartment changes in the comparisons of ECN1 vs. Morex; Table S16: KEGG enrichment of genes with B-to-A compartment changes in the comparisons of ECS1 vs. Morex; Table S17: The annotation of SNP variations in Horvu_MOREX_3H01G165700; Table S18: The annotation of SNP variations in Horvu_MOREX_3H01G166100; Table S19: TAD domain identifications in the three genomes; Table S20: Clustering of TAD boundaries of the three genomes; Table S21. Conversion of 169 CNV complex loci from Morex reference genome v3 to v2.
