# Supplementary material for: Integrated Comparative Genomics and Population Genomics Reveal the Genomic and Epigenomic Basis of Barley (Hordeum vulgare) Evolution and Domestication
Source: Plants (Basel). 2026 Jul 29;15(15):2341. doi: 10.3390/plants15152341 (PMC13467946; doi:10.3390/plants15152341)
Supplement: Supplementary file 1 [file plants-15-02341-s001.zip › plants-4408886-supplementary figures.pdf]

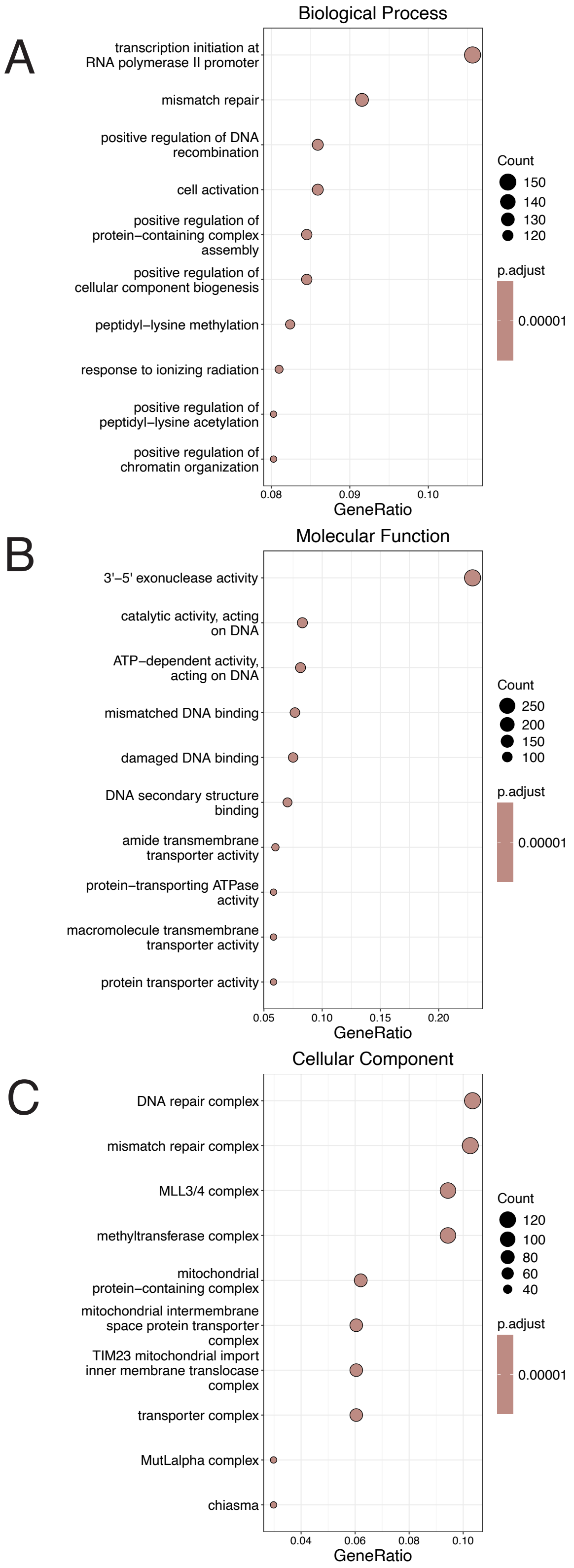

Figure S1. GO enrichment of barley-specific genes. Top 10 enriched terms in biological process (A), molecular functions (B), and cellular component (C) are shown.

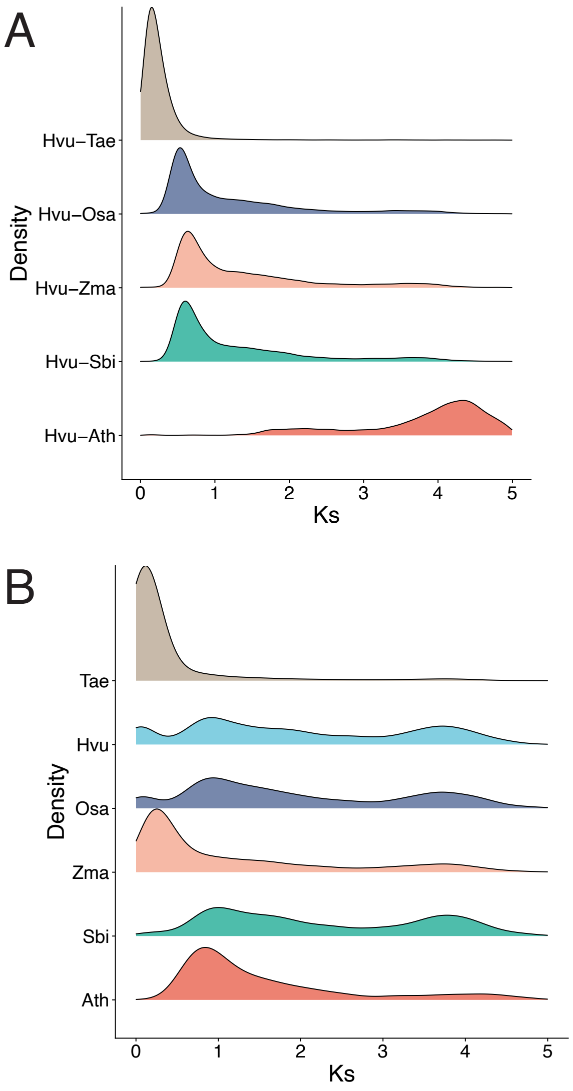

Figure S2. Estimation of plant divergence (A) and whole-genome duplication (B) based on Ks distributions.t Abbreviation: Ath, *Arabidopsis thaliana*; Osa: *Oryza sativa*; Sbi, *Sorghum bicolor*; Zma, *Zea mays*; Hvu, *Hordeum vulgare*; Tae, *Triticum aestivum*.

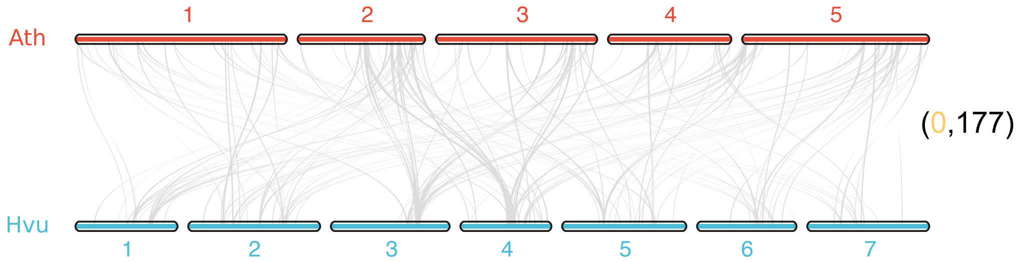

Figure S3. Genomic syntenic analysis between barley (Hvu) and Arabidopsis (Ath). The numbers in the parentheses indicate large (> 200 gene pairs, yellow) and small (5-200 gene pairs, black) syntenic blocks.

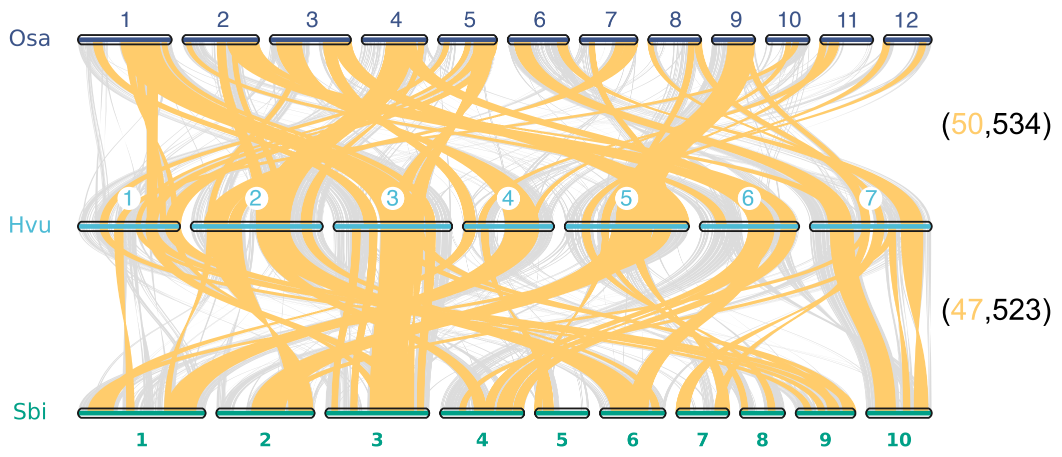

Figure S4. Genomic syntenic analysis between barley (Hvu) and rice (Osa) or sorghum (Sbi). The numbers in the parentheses indicate large (> 200 gene pairs, yellow) and small (5-200 gene pairs, black) syntenic blocks.

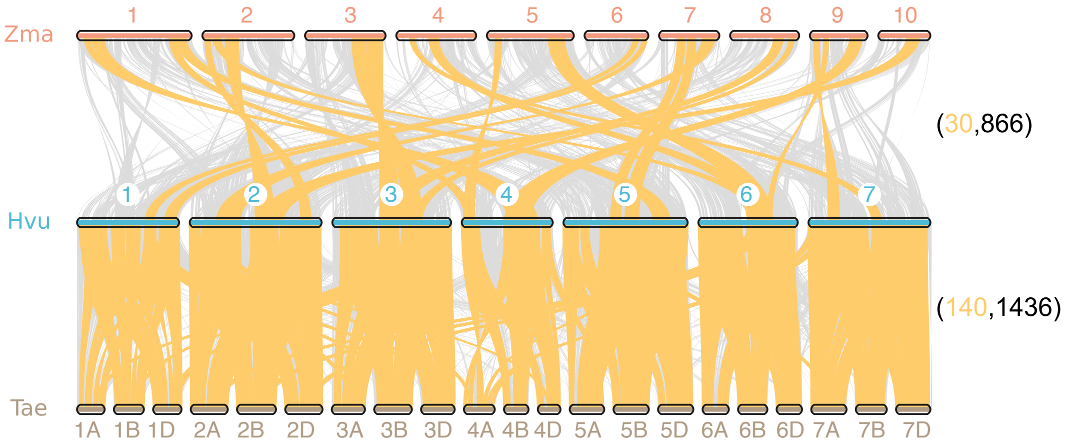

Figure S5. Genomic syntenic analysis between barley (Hvu) and maize (Zma) or wheat (Tae). The numbers in the parentheses indicate large (> 200 gene pairs, yellow) and small (5-200 gene pairs, black) syntenic blocks.

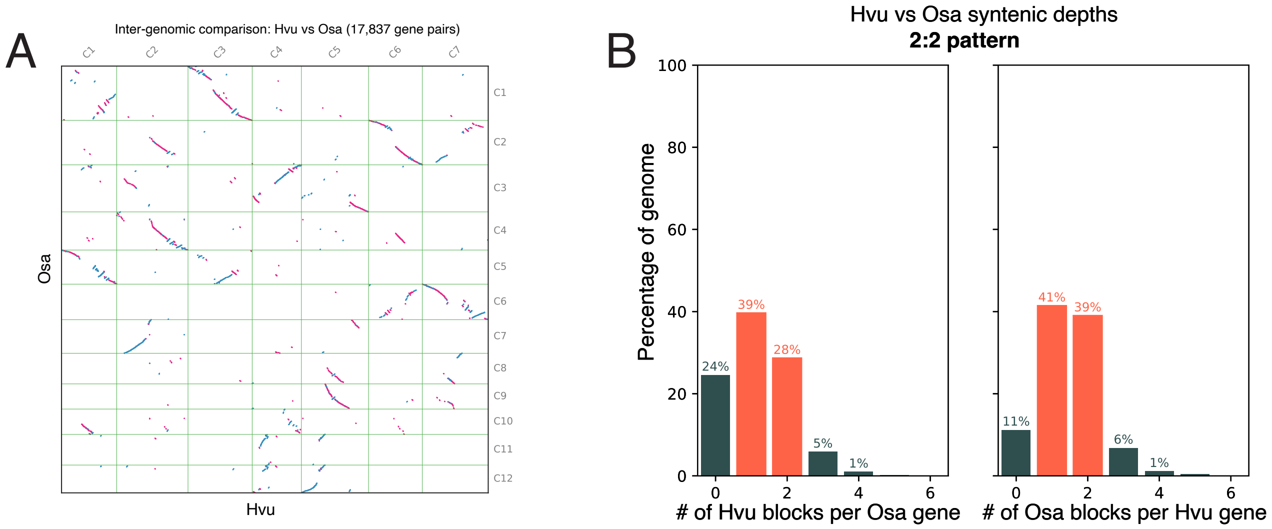

Figure S6. Dot plot analysis (A) and syntenic depth analysis (B) of genome-wide syntenic blocks between barley (Hvu) and rice (Osa).

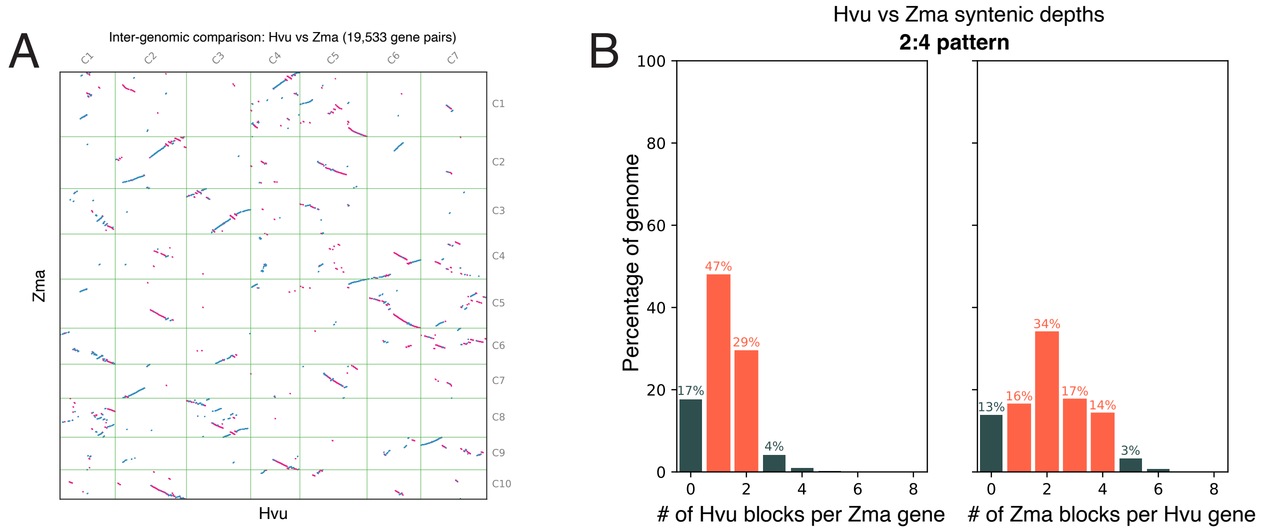

Figure S7. Dot plot analysis (A) and syntenic depth analysis (B) of genome-wide syntenic blocks between barley (Hvu) and maize (Zma).

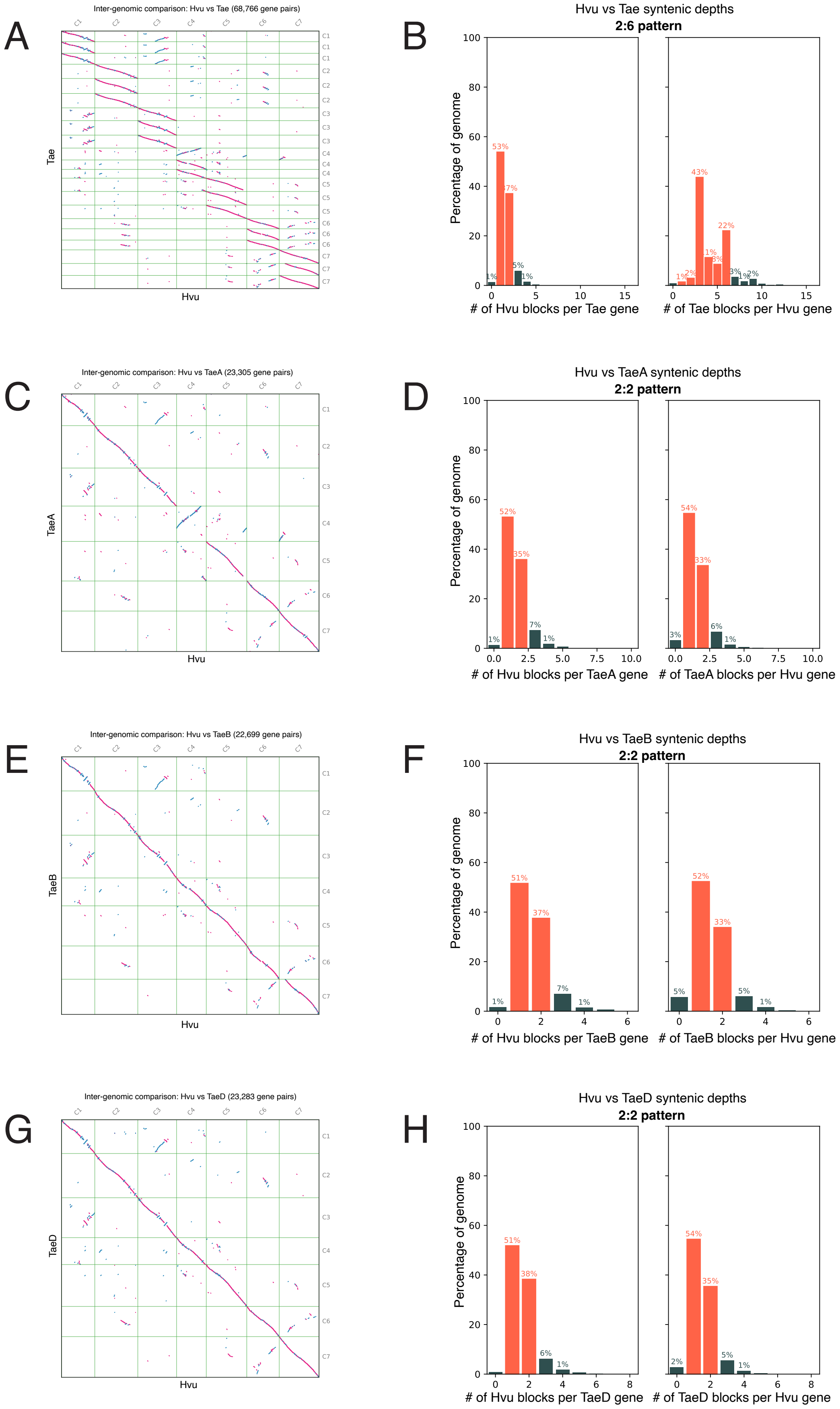

Figure S8. Dot analysis (A, C, E and G) and syntenic depth analysis (B, D, F and H) of genome-wide syntenic blocks between barley (Hvu) and wheat (Tae), wheat sub-genome A (TaeA), wheat sub-genome B (TaeB) or wheat sub-genome D (TaeD).

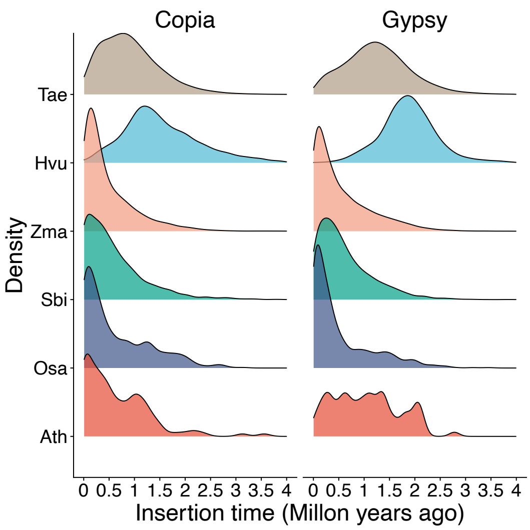

Figure S9. Estimation of insertion times of Copia and Gypsy transposons in different plant species. Abbreviation: Ath, *Arabidopsis thaliana*; Osa: *Oryza sativa*; Sbi, *Sorghum bicolor*; Zma, *Zea mays*; Hvu, *Hordeum vulgare*; Tae, *Triticum aestivum*.

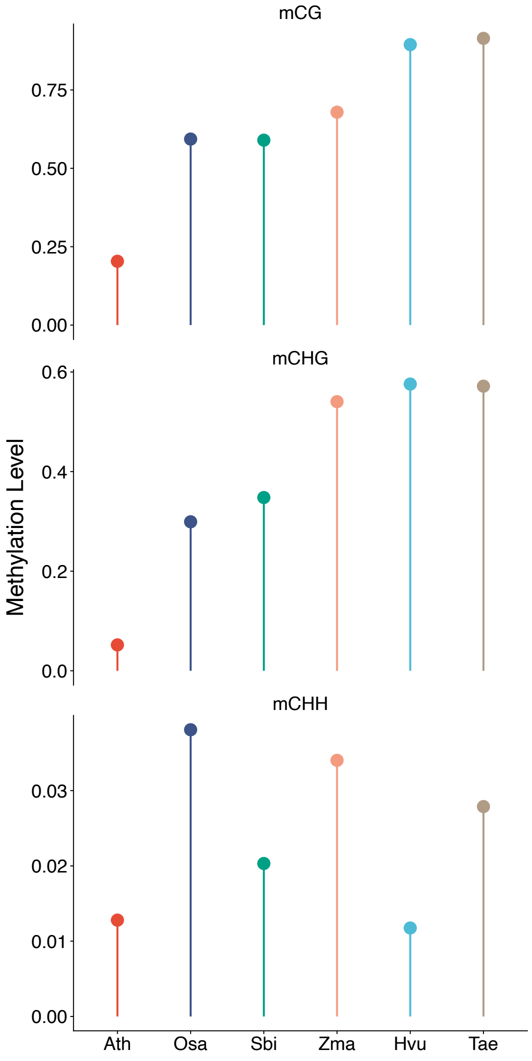

Figure S10. DNA methylation levels in three sequence contexts in different plant genomes. Abbreviation: Ath, *Arabidopsis thaliana*; Osa: *Oryza sativa*; Sbi, *Sorghum bicolor*; Zma, *Zea mays*; Hvu, *Hordeum vulgare*; Tae, *Triticum aestivum*.

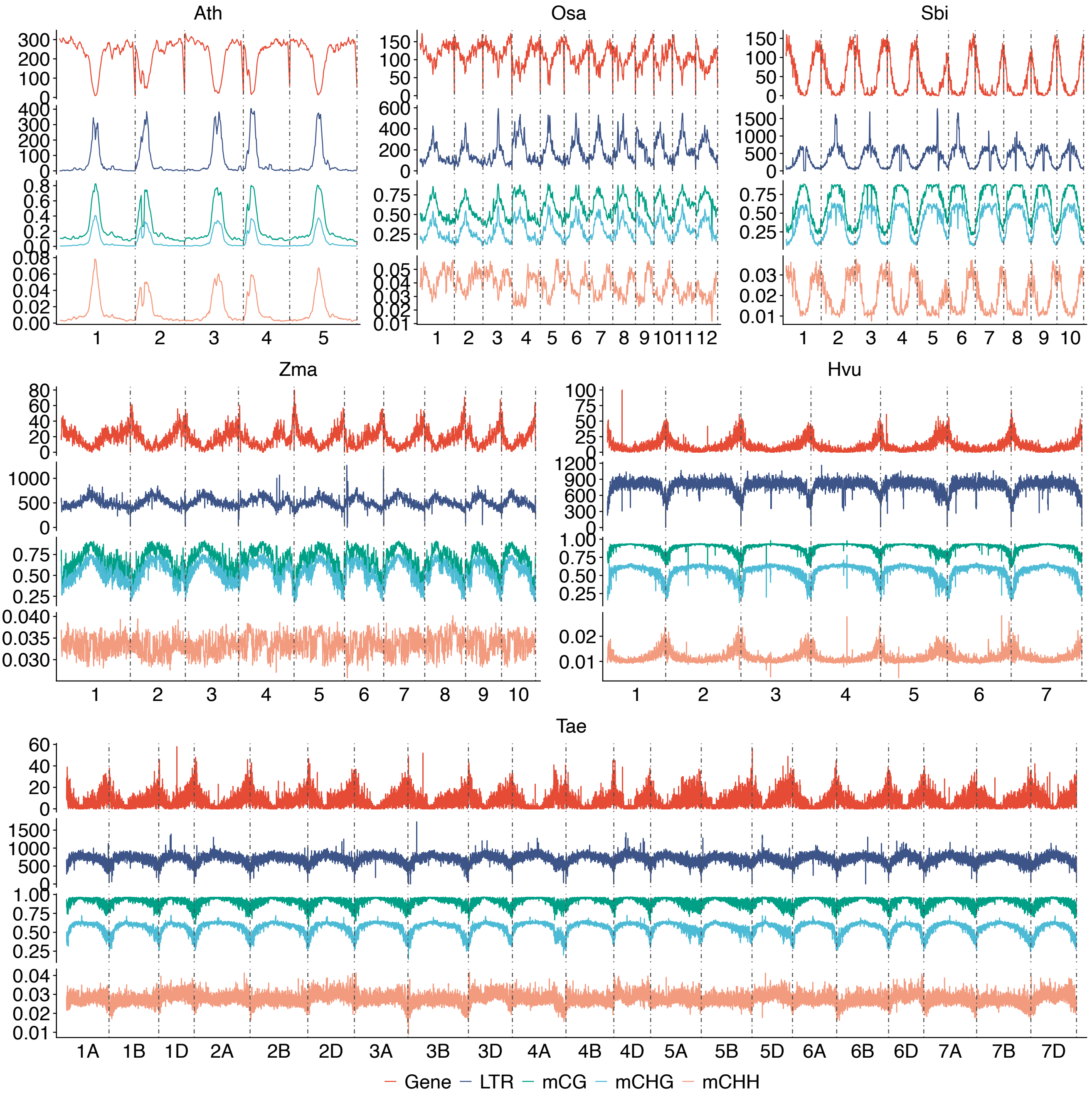

Figure S11. Distributions of genes, LTRs, and DNA methylation levels of mCG, mCHG and mCHH along chromosomes of different plant species. The density is calculated using 1Mb sliding windows with 200 kb steps. Abbreviation: Ath, *Arabidopsis thaliana*; Osa: *Oryza sativa*; Sbi, *Sorghum bicolor*; Zma, *Zea mays*; Hvu, *Hordeum vulgare*; Tae, *Triticum aestivum*.

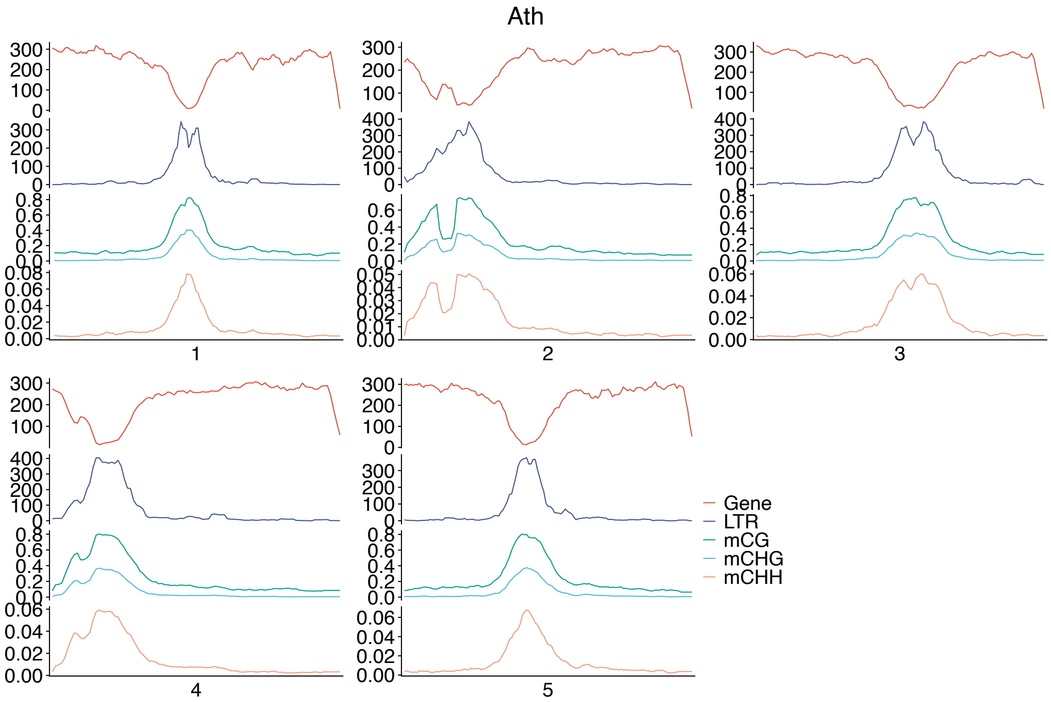

Figure S12. Distributions of genes, LTRs, and DNA methylation levels of mCG, mCHG and mCHH along different chromosomes of Arabidopsis (Ath). The density is calculated using 1Mb sliding windows with 200 kb steps.

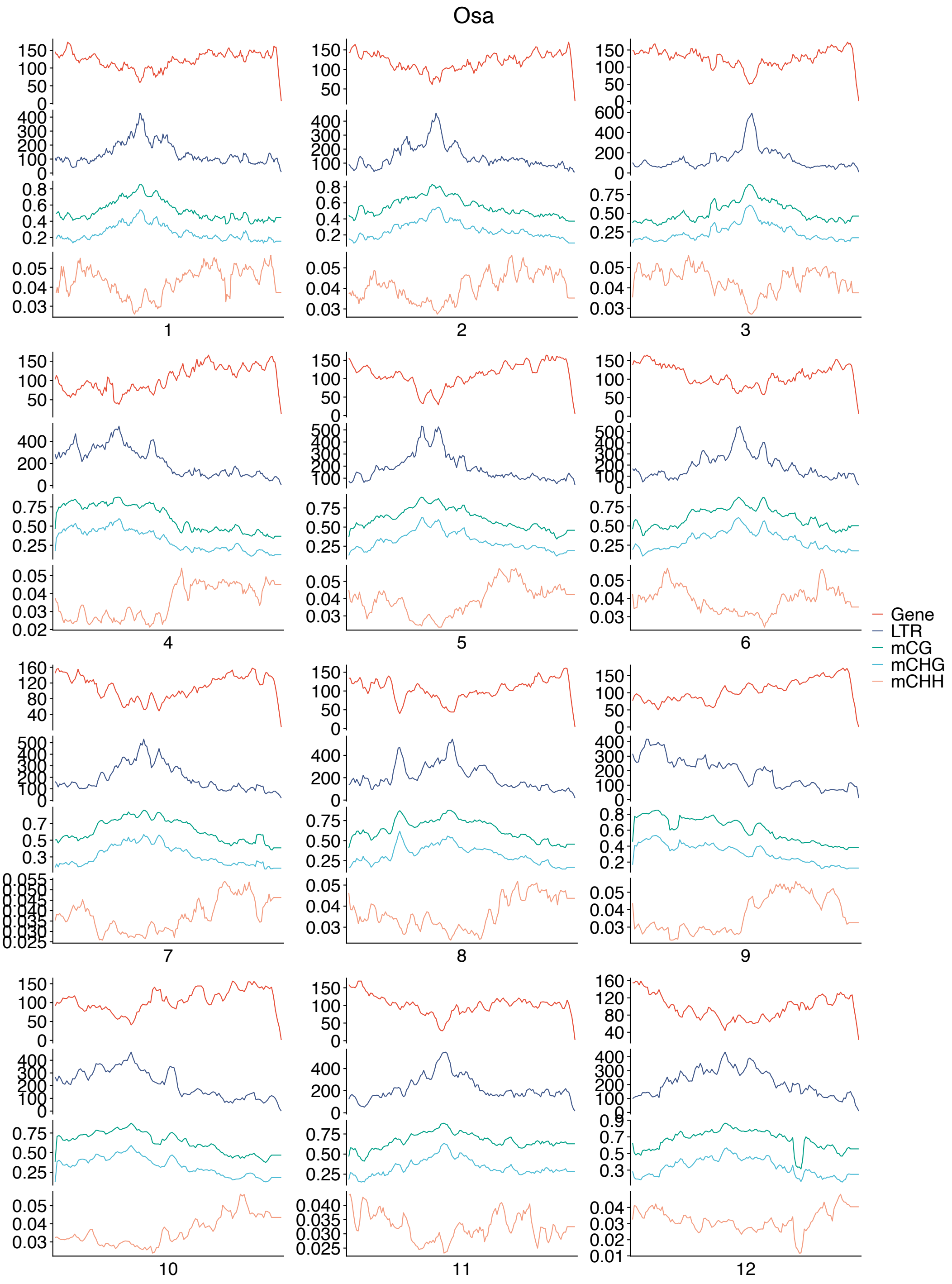

Figure S13. Distributions of genes, LTRs, and DNA methylation levels of mCG, mCHG and mCHH along different chromosomes of rice (Osa). The density is calculated using 1Mb sliding windows with 200 kb steps.

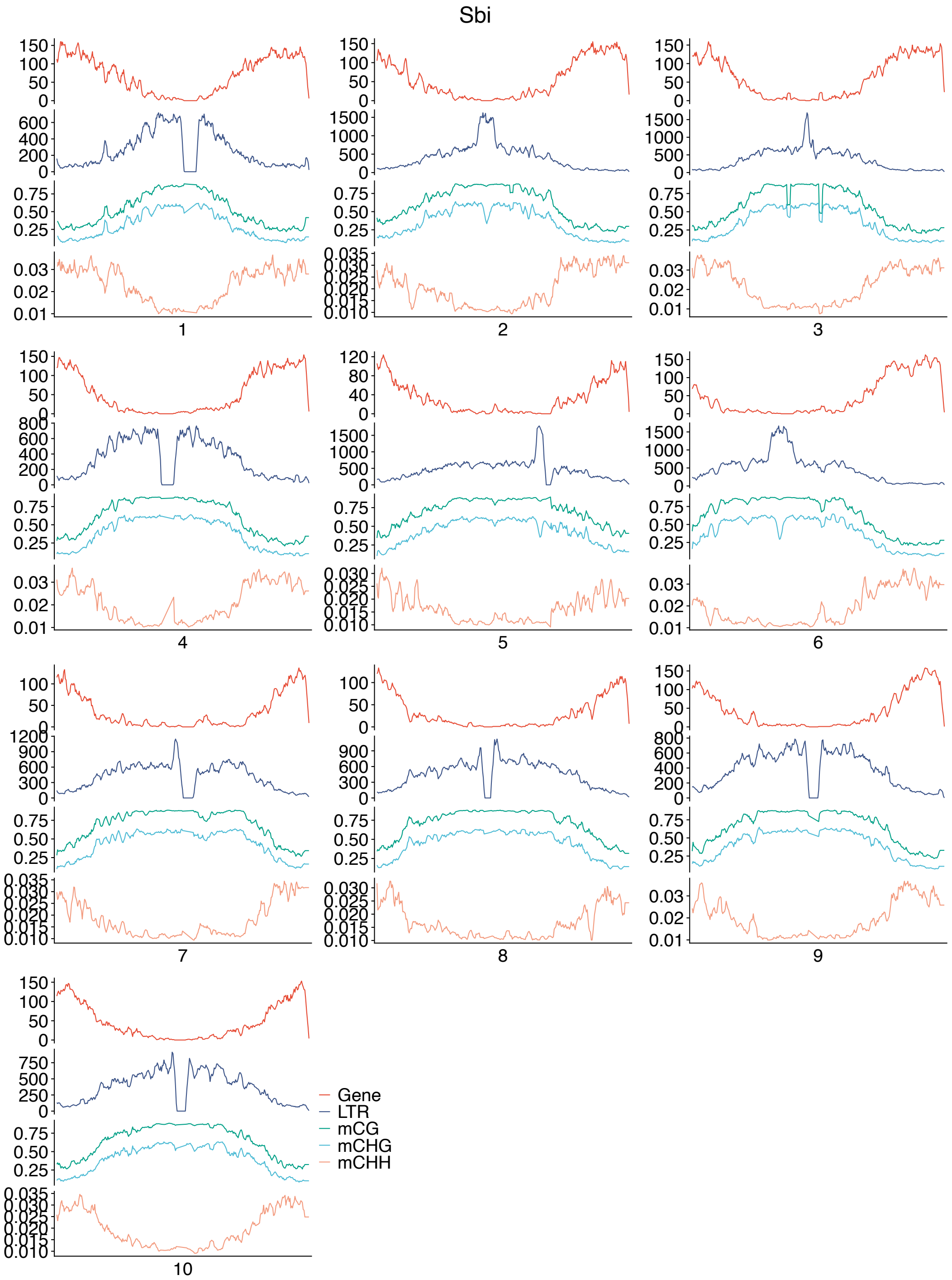

Figure S14. Distributions of genes, LTRs, and DNA methylation levels of mCG, mCHG and mCHH along different chromosomes of sorghum (Sbi). The density is calculated using 1Mb sliding windows with 200 kb steps.

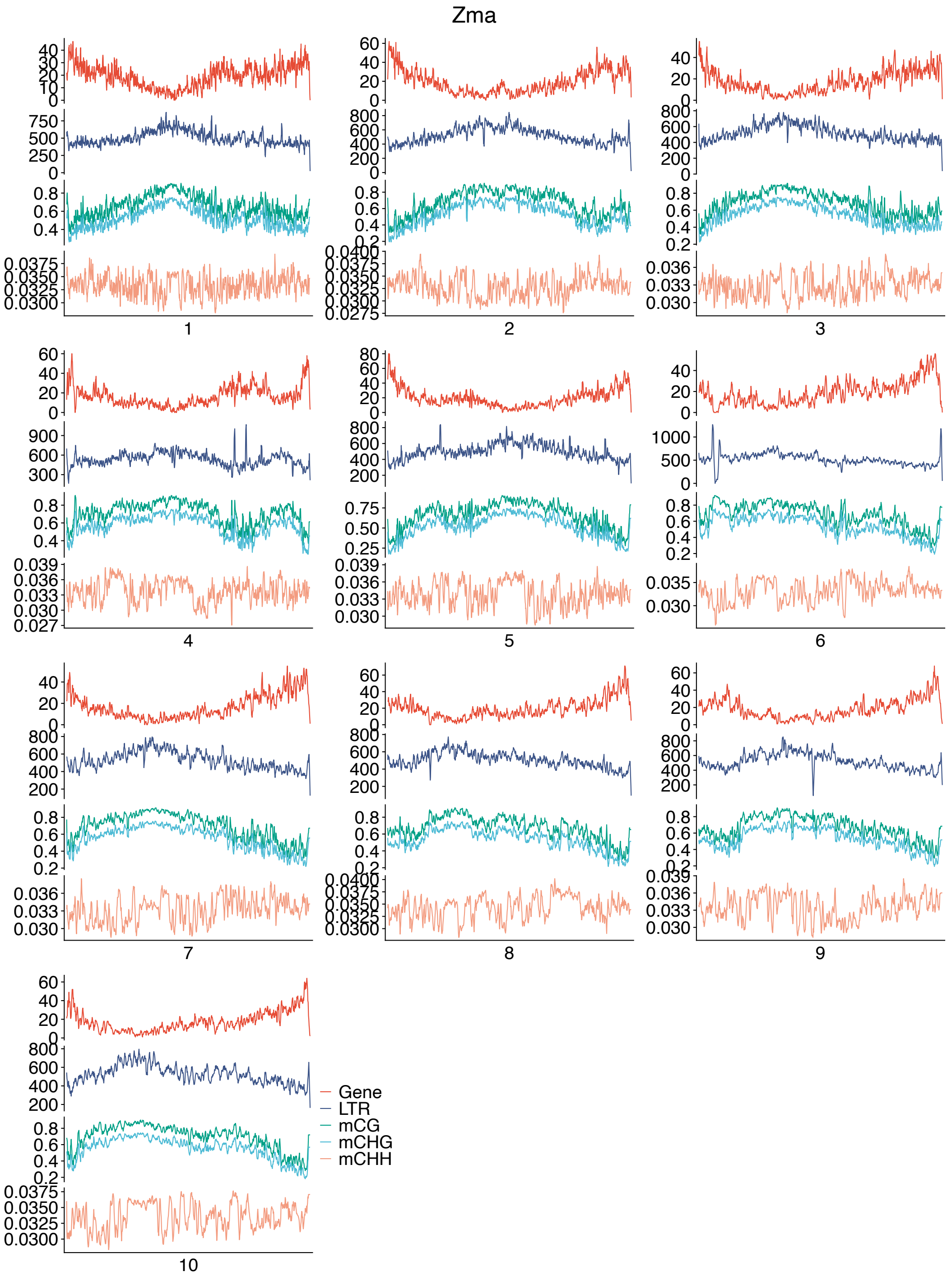

Figure S15. Distributions of genes, LTRs, and DNA methylation levels of mCG, mCHG and mCHH along different chromosomes of maize (Zma). The density is calculated using 1Mb sliding windows with 200 kb steps.

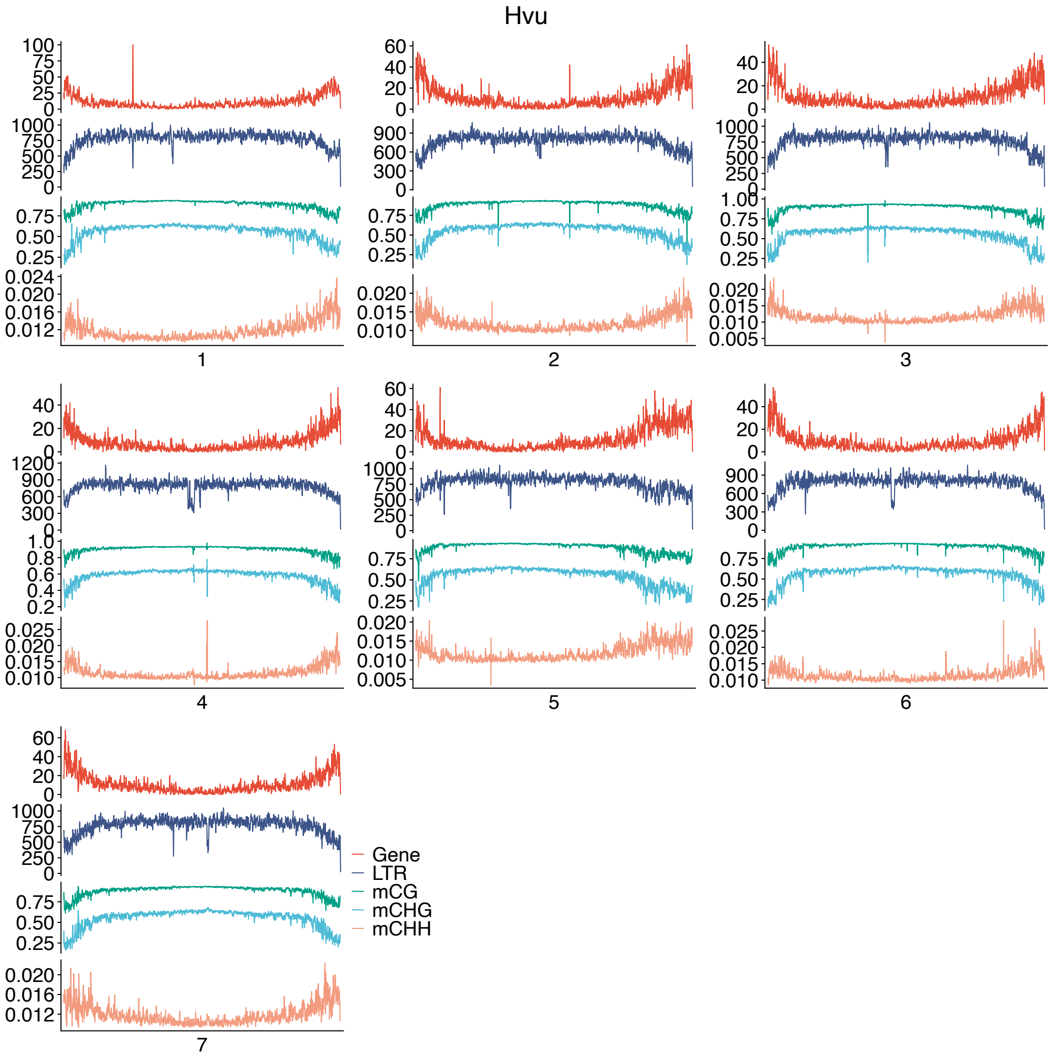

Figure S16. Distributions of genes, LTRs, and DNA methylation levels of mCG, mCHG and mCHH along different chromosomes of barley (Hvu). The density is calculated using 1Mb sliding windows with 200 kb steps.

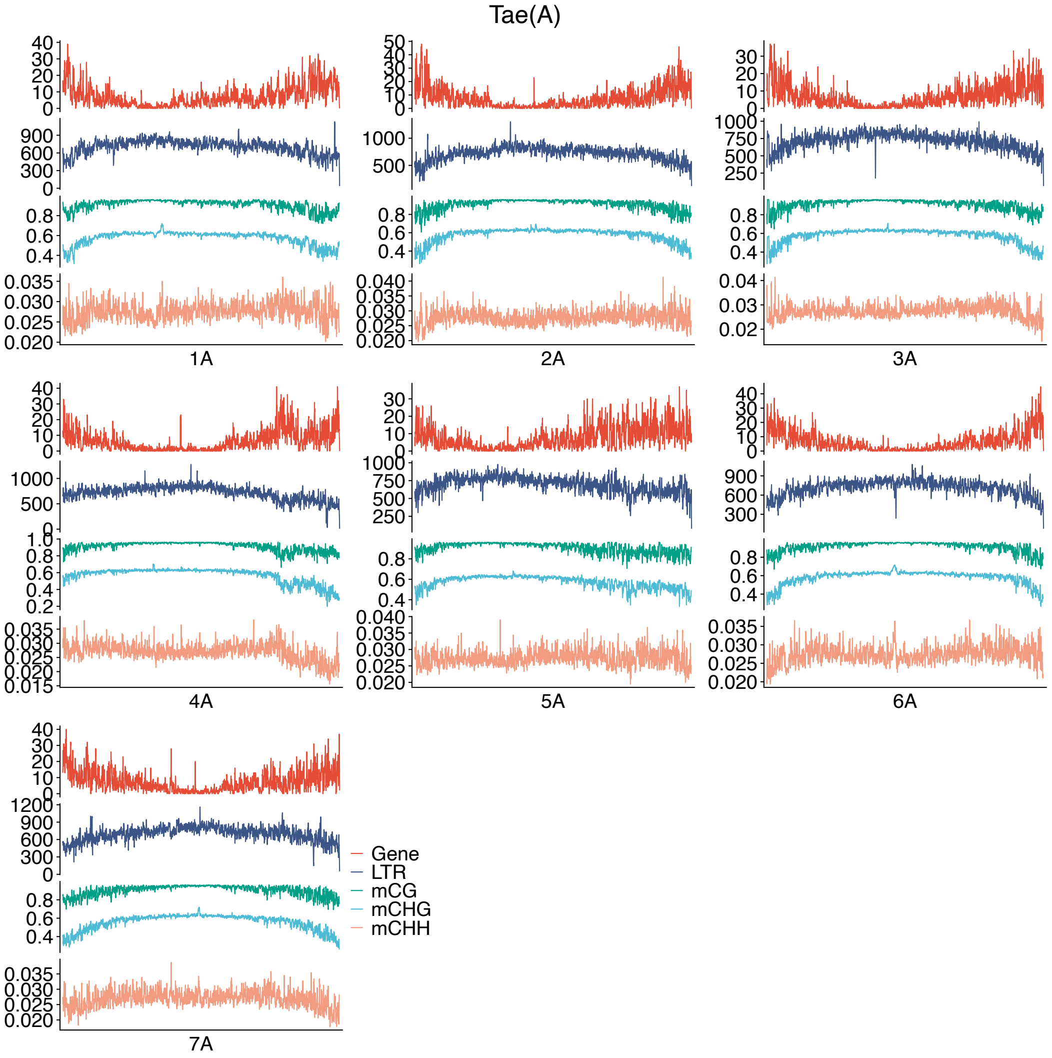

Figure S17. Distributions of genes, LTRs, and DNA methylation levels of mCG, mCHG and mCHH along different chromosomes of wheat sub-genome A (TaeA). The density is calculated using 1Mb sliding windows with 200 kb steps.

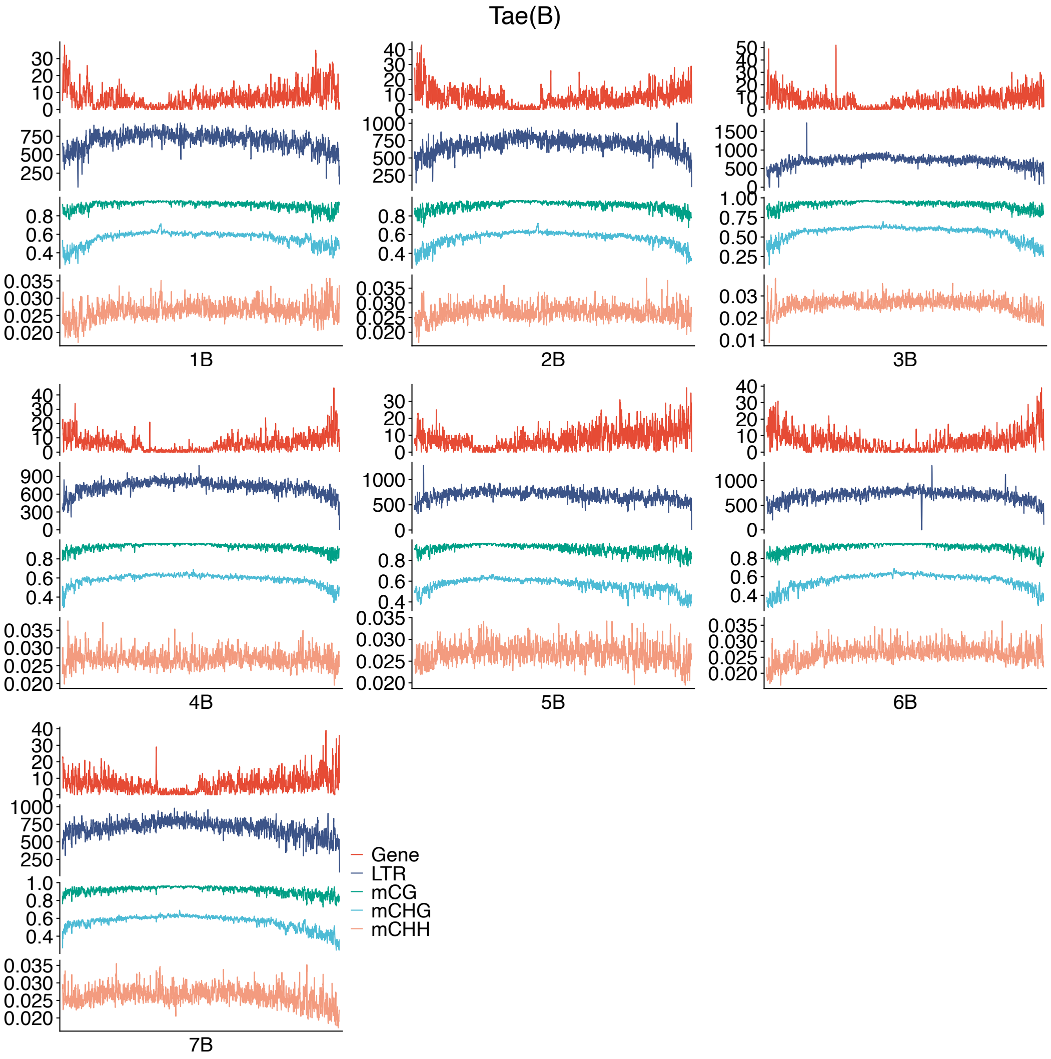

Figure S18. Distributions of genes, LTRs, and DNA methylation levels of mCG, mCHG and mCHH along different chromosomes of wheat sub-genome B (TaeB). The density is calculated using 1Mb sliding windows with 200 kb steps.

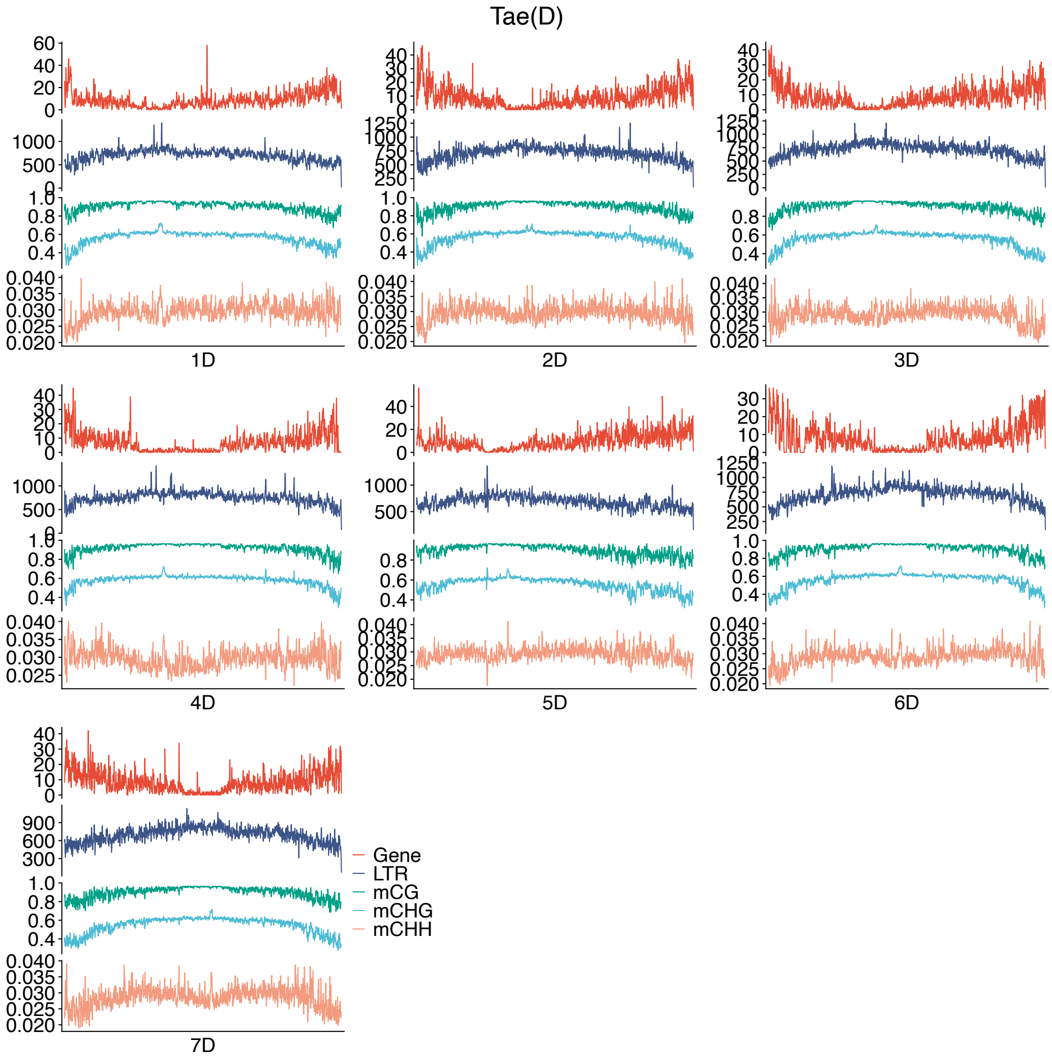

Figure S19. Distributions of genes, LTRs, and DNA methylation levels of mCG, mCHG and mCHH along different chromosomes of wheat sub-genome D (TaeD). The density is calculated using 1Mb sliding windows with 200 kb steps.

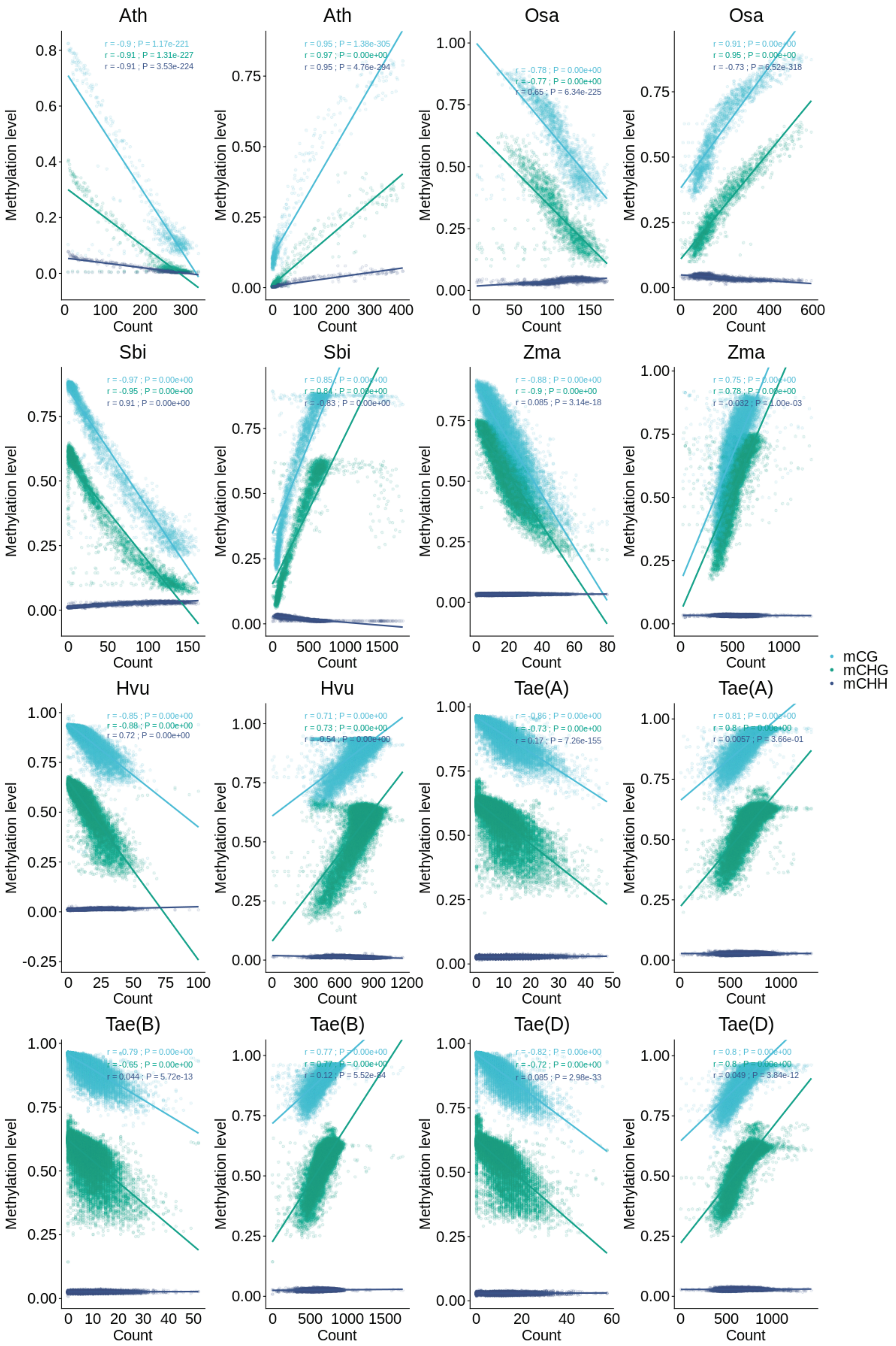

Figure S20. The correlation between methylation levels of mCG, mCHG and mCHH and gene (left panel of each species) or TE density (right panel of each species) using 1Mb sliding windows with 200 kb stepsamong different plant species. Abbreviation: Ath, *Arabidopsis thaliana*; Osa: *Oryza sativa*; Sbi, *Sorghum bicolor*; Zma, *Zea mays*; Hvu, *Hordeum vulgare*; Tae, *Triticum aestivum*.

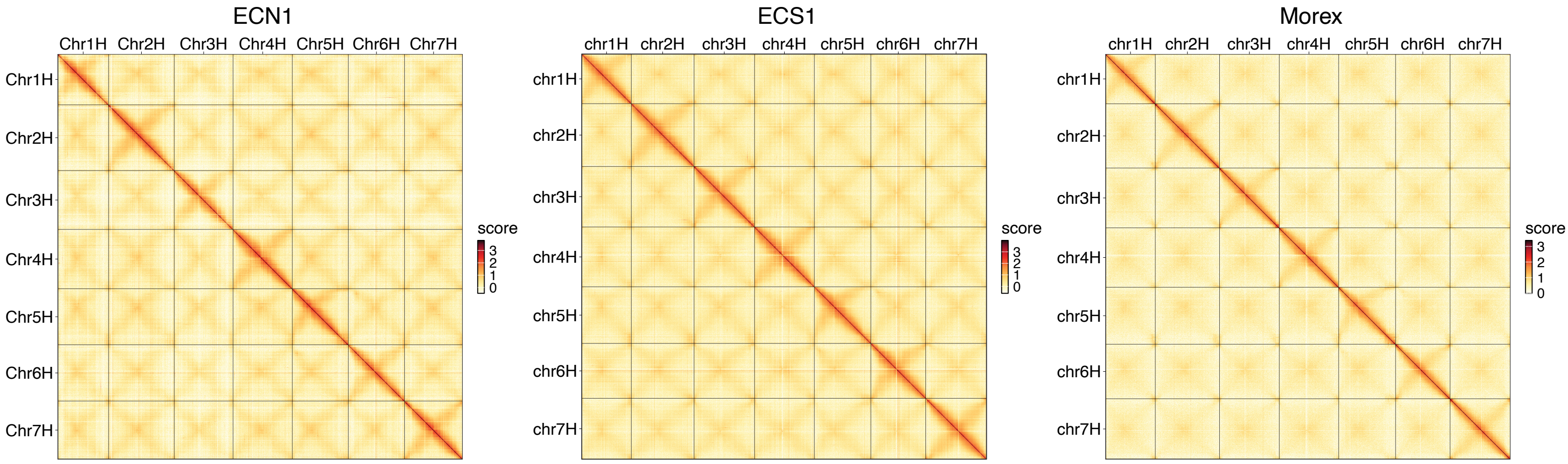

Figure S21. Genome-wide Hi-C contact maps of wild barley (ECN1 and ECS1) and Morex in contiguous 1 Mb windows at the resolution of 70 kb.

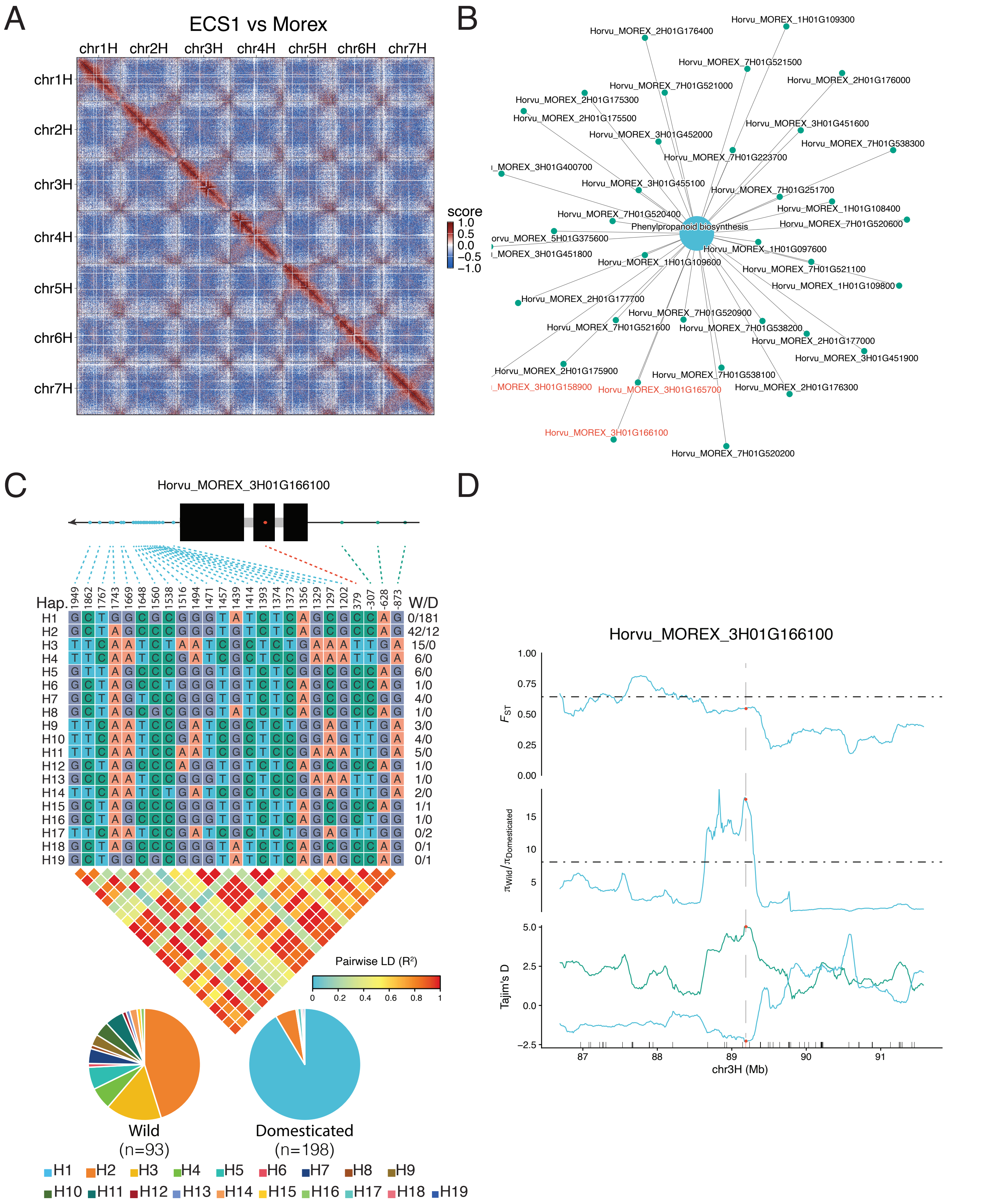

Figure S22. A/B compartment reorganization during barley domestication and selection analysis of a peroxidase-encoding gene Horvu_MOREX_3H01G166100. (A) The genome-wide log2-transformed ratio of normalized and corrected Hi-C contact matrices between ECS1 and Morex in contiguous 1 Mb windows at 70 kb resolution. The red and blue indicate contact enrichment in ECS1 and Morex, respectively. (B) The genes with B-to-A compartment transitions in comparison of ECS1 versus Morex are enriched in phenylpropanoid biosynthesis. Red colors mark the domestication-associated genes. (C) The haplotype and genotype frequency analysis of Horvu_MOREX_3H01G166100 during barley domestication. The SNPs in the upstream, CDS, intron and downstream are marked in green, red, black and blue colors, respectively, labeled with relative positions to the gene start positions. The gene direction in the barley reference genome is indicated with a black arrow. (D) The F_{ST}, π and Tajima's D values within the 5Mb genomic region around the Horvu_MOREX_3H01G166100. The dashed horizontal and vertical lines denote the genome-wide threshold values (top 1%) of selection signals (F_{ST}: 0.64; π: 8.04) and the gene position, respectively. The vertical lines in the x-axis indicate the genes within this region with Horvu_MOREX_3H01G166100 marked in red color.

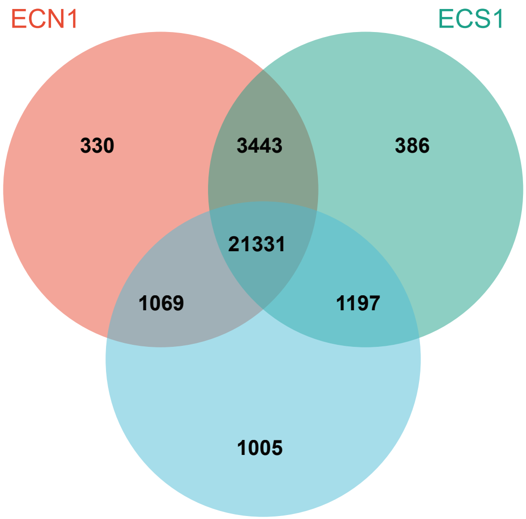

Figure S23. Gene family clustering analysis among the genomes of wild barley (ECN1 and ECS1) and Morex.
